# Synergistic Promotion of Triple‐Negative Breast Cancer Tumorigenesis and Metastasis by Oral Polystyrene Nanoplastics Exposure via *Alloprevotella*‐Derived Glutamate and Platelet Activation

**DOI:** 10.1002/advs.202508310

**Published:** 2025-09-24

**Authors:** Leilei Zhu, Peihao Xu, Mingyuan Zhou, Kewei Li, Shasha Tian, Xuemei Fan, Junling Liu, Baodong Ye, Zhishan Ding, Yingzhi Shen

**Affiliations:** ^1^ School of Medical Technology and Information Engineering The First Affiliated Hospital of Zhejiang Chinese Medical University (Zhejiang Provincial Hospital of Chinese Medicine) Zhejiang Chinese Medical University Hangzhou Zhejiang 310053 China; ^2^ School of Pharmaceutical Sciences Zhejiang Chinese Medical University Hangzhou Zhejiang 310053 China; ^3^ Department of Biochemistry and Molecular Cell Biology Shanghai Jiao Tong University School of Medicine Shanghai 200025 China; ^4^ Department of Hematology The First Affiliated Hospital of Zhejiang Chinese Medical University (Zhejiang Provincial Hospital of Chinese Medicine) Hangzhou Zhejiang 310053 China

**Keywords:** *Alloprevotella*, glutamate, platelet, polystyrene nanoplastics, triple‐negative breast cancer

## Abstract

Nanoplastics (NPs) are emerging environmental pollutants with potential health risks, yet their role in cancer progression remains poorly understood. Here, this is demonstrated that oral exposure to 100 nm polystyrene nanoplastics (PS‐NPs) accelerates tumor initiation and metastasis in a murine triple‐negative breast cancer (TNBC) model, without affecting primary tumor growth. PS‐NPs do not directly alter TNBC cell behavior in vitro but induced gut microbiota dysbiosis, characterized by *Alloprevotella* enrichment and elevated systemic glutamate levels, both identified as key mediators of PS‐NPs‐driven tumor promotion. Moreover, PS‐NPs enhanced platelet activation, evidenced by increased aggregation, microthrombus formation at metastatic sites, and upregulation of CD36 and Serpine1. Collectively, these findings uncover a synergistic mechanism whereby oral PS‐NPs promote TNBC progression via a gut microbiota‐derived metabolite‐platelet axis, establishing an unrecognized link between environmental nanoplastic exposure and cancer progression, and highlighting potential therapeutic targets for intervention.

## Graphical abstract

Polystyrene nanoplastics (PS‐NPs) administered via oral exposure accelerate triple‐negative breast cancer (TNBC) progression by synergistically modulating gut microbiota‐derived glutamate production and platelet activation. This study uncovers a pivotal crosstalk between microbial metabolites and platelet‐tumor interactions, offering mechanistic insights into systemic perturbations induced by environmental pollutants and highlighting potential therapeutic targets for intervention.

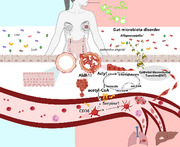

## 1 Introduction

Triple‐negative breast cancer (TNBC), the most aggressive subtype of breast cancer, accounts for 15%–20% of all cases and is characterized by a high propensity for invasion, metastasis, and therapeutic resistance.^[^ ^1^ ^]^ Defined by the absence of estrogen receptor (ER), progesterone receptor (PR), and human epidermal growth factor receptor 2 (HER2), TNBC also exhibits marked intratumoral heterogeneity.^[^ ^2^ ^]^ Although considerable progress has been made in delineating its molecular landscape, the contribution of environmental factors to TNBC progression remains poorly understood. Epidemiological and experimental evidence implicates environmental toxicants, including chemical pesticides, heavy metals, and polycyclic aromatic hydrocarbons, as significant risk factors for breast cancer initiation and progression.^[^ ^3^, ^4^, ^5^ ^]^

Nanoplastics (NPs), plastic particles less than 1 µm in diameter, enter the human body via ingestion, inhalation, or dermal absorption, and can traverse biological barriers such as the vascular endothelium, blood‐testis barrier, and placenta.^[^ ^6^ ^]^ Accumulating studies link NPs to reproductive toxicity, cardiovascular dysfunction, and carcinogenesis.^[^ ^7^, ^8^ ^]^ Due to their nanoscale dimensions and high surface area‐to‐volume ratio, NPs display distinct bioactivity, inducing oxidative stress, DNA damage, and perturbations of gut microbial homeostasis, processes that may facilitate tumorigenesis and progression.^[^ ^9^, ^10^, ^11^ ^]^ Among various NPs types, polystyrene nanoplastics (PS‐NPs) have drawn particular concern for their oncogenic potential. PS‐NPs disrupt lipid metabolism and remodel gut microbiota, thereby promoting colorectal tumorigenesis and ovarian cancer progression,^[^ ^12^, ^13^ ^]^ making them a representative model for investigating the carcinogenic effects of environmental nanoplastics.

Oral nanoparticle exposure has been shown to disturb gut microbial homeostasis, reducing commensal taxa and expanding pathogenic populations, thereby impairing intestinal barrier integrity.^[^ ^10^, ^14^ ^]^ The gut microbiota also regulates platelet activation through bile acid‐dependent signaling in a microbially modulated manner.^[^ ^15^ ^]^ Both microbial dysbiosis and platelet activation are independently associated with tumor progression.^[^ ^16^, ^17^ ^]^ These observations raise the possibility that nanoplastic ingestion may promote TNBC tumorigenesis and metastasis through coordinated modulation of gut microbiota and platelet function.

While previous studies have demonstrated that nanoplastics can alter gut microbiota, activate platelets, or enhance tumor progression individually, whether they drive TNBC malignancy via an integrated gut microbiota‐platelet axis remains unknown.^[^ ^18^, ^19^ ^]^ Here, we show that oral PS‐NPs exposure enriches *Alloprevotella* in the gut and increases its glutamate production, leading to systemic disruption and platelet activation. These changes collectively enhance breast tumor stemness, epithelial‐mesenchymal transition (EMT), and ATP‐citrate lyase (Acly) ‐dependent fatty acid biosynthesis, thereby promoting TNBC initiation and metastasis. Our findings reveal a mechanistic link between PS‐NPs ingestion and TNBC progression and identify a gut microbiota‐metabolite‐platelet axis as a potential therapeutic target for mitigating the oncogenic impact of environmental nanoplastics.

## 2 Results

### 2.1 Oral PS‐NPs Exposure Promotes TNBC Initiation Without Affecting Primary Tumor Growth

To evaluate the biological effects of PS‐NPs on TNBC, we utilized commercially available 100‐nm polystyrene microspheres conjugated with green fluorescent protein (GFP) for in situ visualization. The particles formed a green suspension and exhibited uniform morphology under scanning electron microscopy (SEM) (Figure S1a, Supporting Information). For oral administration, PS‐NPs were ultrasonically dispersed in sterile water and provided ad libitum, with solutions replaced every two days to ensure dispersion stability (Figure S1b, Supporting Information).

**Figure 1:**
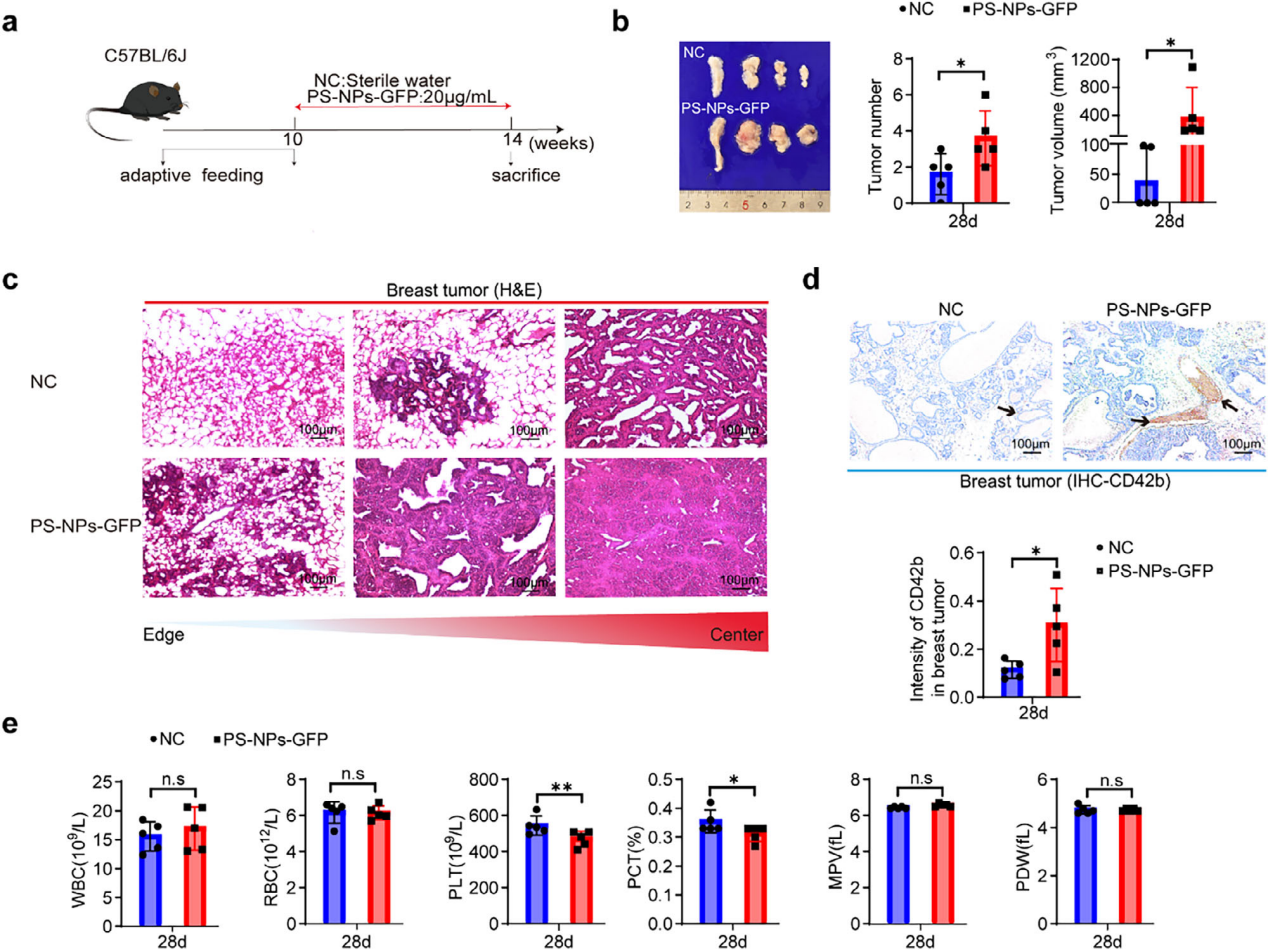
Oral PS‐NPs exposure promoted TNBC initiation and reduced peripheral platelet counts in *MMTV‐PyMT* mice. a), Experimental design. Ten‐week‐old *MMTV‐PyMT* transgenic mice were administered PS‐NPs‐GFP (20 µg mL^−1^) in drinking water ad libitum for 4 weeks prior to sacrificed (n = 5 per group). b), Gross morphology of mammary tumors from representative mice in the NC and PS‐NPs‐GFP groups. Tumor number and tumor volume (calculated as volume = a × b^2^ × 0.5236, where a is the longest diameter and b is the shortest diameter) per mouse were significantly increased in the PS‐NPs‐GFP group compared with NC (mean ± SEM; n = 5 per group). c), Representative hematoxylin and eosin (H&E)‐stained sections of mammary tumors from each group at endpoint, showing histopathological features associated with tumor progression (n = 5 per group). d), Representative IHC images of tissue microarrays containing breast tumor samples, with quantification of CD42b staining scores (mean ± SEM; n = 5 per group). e), Peripheral blood analysis of white blood cell (WBC), red blood cell (RBC), platelet (PLT), and platelet indices, including platelet distribution width (PDW), mean platelet volume (MPV), and plateletcrit (PCT) (mean ± SEM; n = 5 per group). Data in (b,d,e) were analyzed by unpaired two‐tailed t‐tests. *p < 0.05; **p < 0.01; n.s, not significant.

In the *MMTV‐PyMT* transgenic model, which spontaneously develops multifocal mammary tumors, 10‐week‐old female mice received PS‐NPs (20 µg mL^−1^; estimated intake ≈0.1 mg/mouse/day) or sterile water for four weeks. Baseline water consumption was comparable between groups (**Figure** **1**a; Figure S1c, Supporting Information). PS‐NPs exposure significantly increased tumor burden, as reflected by higher tumor numbers and volumes (Figure 1b). Histological analysis revealed accelerated progression from ductal carcinoma in situ to invasive carcinoma, with a malignancy gradient from the periphery to the tumor core. Immunohistochemical staining for CD42b demonstrated greater intratumoral platelet aggregation and more extensive platelet‐rich thrombi in mammary tumors of PS‐NPs‐treated mice (Figure 1c,d). In vivo GFP tracing confirmed PS‐NPs accumulation in the intestinal tract, with no evident intestinal injury (Figure S1d,e, Supporting Information).

Pyrolysis‐gas chromatography‐mass spectrometry (Py‐GC/MS) detected multiple microplastics (MPs) in mammary tumor tissues, including polystyrene (PS), polyethylene (PE), polyethylene terephthalate (PET), styrene‐butadiene rubber (SBR), polyvinyl chloride (PVC), acrylonitrile‐butadiene‐styrene copolymer (ABS), nylon 6 (N6), polyurethane (PU), polycarbonate (PC), and nylon 66 (N66) (Figure S1f, Supporting Information). Unexpectedly, PS levels were lower in tumors from PS‐NPs‐exposed mice compared with controls, while other MPs displayed inconsistent patterns. These observations underscore the current methodological challenges in accurately quantifying MPs in tumor tissues, where environmental background signals, heterogeneous tumor composition, and analytical sensitivity complicate interpretation. Therefore, our study primarily focuses on the indirect, systemic pro‐tumorigenic effects of PS‐NP exposure rather than their direct accumulation within tumors.

Peripheral blood analysis showed no changes in leukocyte or erythrocyte counts following PS‐NPs exposure. However, platelet count (PLT) and plateletcrit (PCT) were significantly reduced, whereas platelet distribution width (PDW) and mean platelet volume (MPV) remained unchanged (Figure 1e), suggesting decreased platelet abundance without altered size distribution. This implicates platelets as potential cellular mediators of PS‐NPs‐induced tumor initiation.

To further examine effects on tumor growth, an orthotopic TNBC model was established by transplanting syngeneic 4T1 cells into the mammary fat pads of BALB/c mice. After tumor establishment, mice were randomized by tumor volume and exposed to PS‐NPs (Figure S2a, Supporting Information). No significant differences in tumor volume or histopathology were observed between groups (Figure S2b,c, Supporting Information). Consistent with the spontaneous model, PS‐NPs localized to the intestinal tract without intestinal pathology (Figure S2d,e, Supporting Information).

Collectively, these results demonstrate that dietary PS‐NPs exposure promotes TNBC initiation in vivo but does not affect the growth of established tumors, likely through indirect mechanisms involving platelet dysregulation.

### 2.2 Oral PS‐NPs Exposure Facilitates Metastasis in TNBC Models

**Figure 2:**
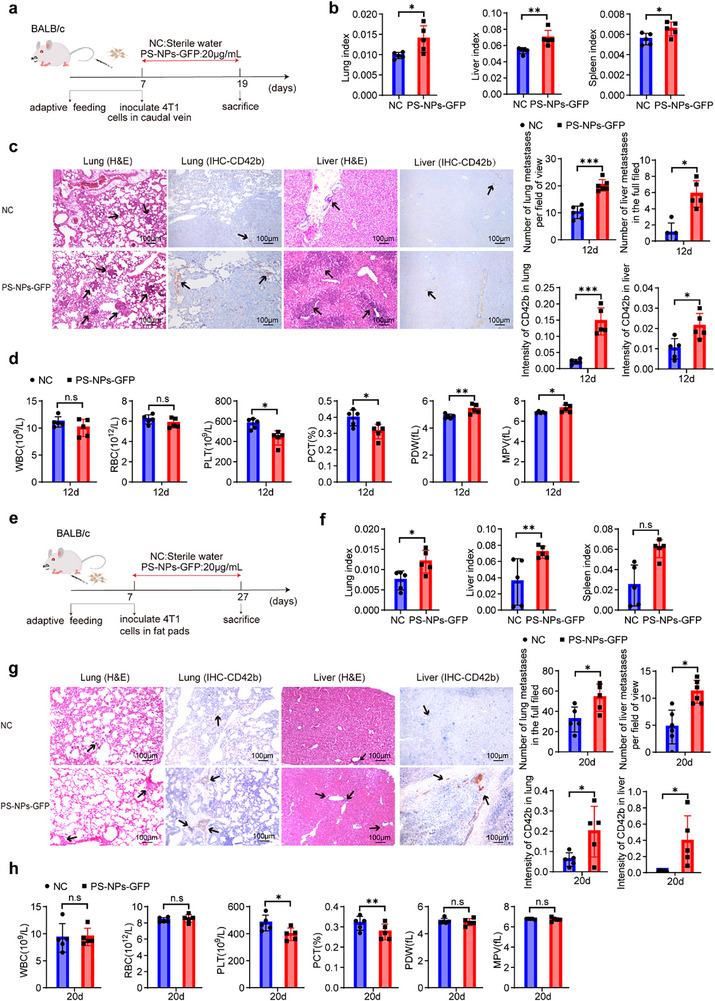
In vivo evaluation of PS‐NPs exposure in promoting TNBC metastasis using caudal vein and fat pad injection models. a), Experimental design for the caudal vein injection model. Seven‐week‐old BALB/c mice were administered PS‐NPs‐GFP (20 µg mL≈) in drinking water ad libitum, followed by caudal vein injection of 4T1 cells. Mice were sacrificed 12 days post‐injection (n = 5 per group). b), Organ index (organ weight/body weight, %) of the lung, liver, and spleen in control and PS‐NPs‐GFP groups (mean ± SEM; n = 5 per group). c), Quantification of metastatic foci in the lungs and livers based on H&E staining. Representative IHC images of tissue microarrays containing lung and liver sections, with quantification of CD42b staining scores (mean ± SEM; n = 5 mice per group). d), Peripheral blood analysis of WBC, RBC, and PLT counts, along with platelet indices (PDW, MPV, PCT) (mean ± SEM; n = 5 per group). e), Experimental design for the fat pad injection model. Seven‐week‐old BALB/c mice received PS‐NPs‐GFP (20 µg mL^−1^) in drinking water and were injected with 4T1 cells into the mammary fat pad. Mice were sacrificed 20 days post‐injection (n = 5 per group). f), Organ index of the lung, liver, and spleen in control and PS‐NPs‐GFP groups (mean ± SEM; n = 5 per group). g), Quantification of metastatic foci in the lung and liver tissues based on H&E staining. Representative IHC images of lung and liver tissue microarrays, with quantification of CD42b staining scores (mean ± SEM, n = 5 mice per group). h), Peripheral blood analysis as in d (mean ± SEM; n = 5 per group). Statistical analysis in b‐d and f‐h was performed using unpaired two‐tailed t‐tests. *p < 0.05; **p < 0.01; ***p < 0.001; n.s, not significant.

TO investigate the impact of PS‐NPs on TNBC metastasis, we first employed a tail vein injection model to mimic hematogenous dissemination (**Figure** **2**a). Mice exposed to PS‐NPs displayed significantly increased liver, lung, and spleen indices compared to controls (Figure 2b), indicating an elevated metastatic burden in the liver and lungs, as well as possible perturbations in hematopoietic function. Histological examination confirmed a marked increase in hepatic and pulmonary metastatic lesions in PS‐NPs‐treated mice. Given the observed alterations in platelet parameters and thrombotic features within metastatic lesions, CD42b immunostaining was performed. PS‐NPs exposure markedly increased platelet‐rich thrombi within metastatic foci in both liver and lung tissues (Figure 2c).

Peripheral blood analysis revealed a significant reduction in platelet count, accompanied by evidence of enhanced platelet activation (Figure 2d). Flow cytometry confirmed this finding, showing increased surface expression of CD62P and Annexin V on circulating platelets following PS‐NPs exposure (Figure S3a, Supporting Information), implicating platelets as mediators of PS‐NPs‐induced hematogenous metastasis during advanced disease stages.

To validate these results, we established an orthotopic spontaneous metastasis model by implanting 4T1 cells into the mammary fat pad (Figure 2e). Consistent with the tail vein injection findings, PS‐NPs exposure significantly elevated liver and lung indices (Figure 2f). In this model, metastatic lesions were predominantly hepatic, often localized near vascular regions, with limited pulmonary involvement. CD42b staining confirmed extensive platelet thrombus formation within liver and lung metastases (Figure 2g), while complete blood counts reinforced the platelet‐mediated nature of this process (Figure 2 h). Similarly, orthotopic implantation of Py8119 cells into the mammary fat pad of C57BL/6J mice confirmed that PS‐NPs exposure promoted hepatic and pulmonary metastases, accompanied by enhanced platelet thrombosis (Figure S3b,c, Supporting Information). Across all models, PS‐NPs were confined to the intestinal tract (Figure S3d, Supporting Information), with no evident intestinal pathology (Figure S3e, Supporting Information).

To assess whether PS‐NPs directly influence tumor cell behavior, 4T1 cells were exposed to varying concentrations of PS‐NPs in vitro. Cell Counting Kit‐8 (CCK‐8), wound healing, and transwell assays showed no dose‐dependent increase in proliferation, migration, or invasion; instead, PS‐NPs elicited mild cytotoxic effects (Figure S4a—c, Supporting Information).

Together, these findings indicate that oral PS‐NPs exposure facilitates TNBC metastasis through indirect mechanisms involving platelet dysregulation, rather than direct tumor cell stimulation. This process is associated with reduced platelet counts, enhanced platelet activation, and formation of platelet‐rich thrombi at metastatic sites.

### 2.3 Oral PS‐NPs Exposure Alters Gut Microbiota Composition and Induces Microbial Dysbiosis During TNBC Progression

The gut microbiota, the largest microbial community in the host, plays a pivotal role in maintaining metabolic homeostasis, while its disruption by environmental factors can promote tumor progression.^[^ ^20^ ^]^ To determine whether oral PS‐NPs exposure modulates gut microbiota during TNBC development, 16S rRNA sequencing was performed on fecal samples from tumorigenesis and metastasis models.

**Figure 3:**
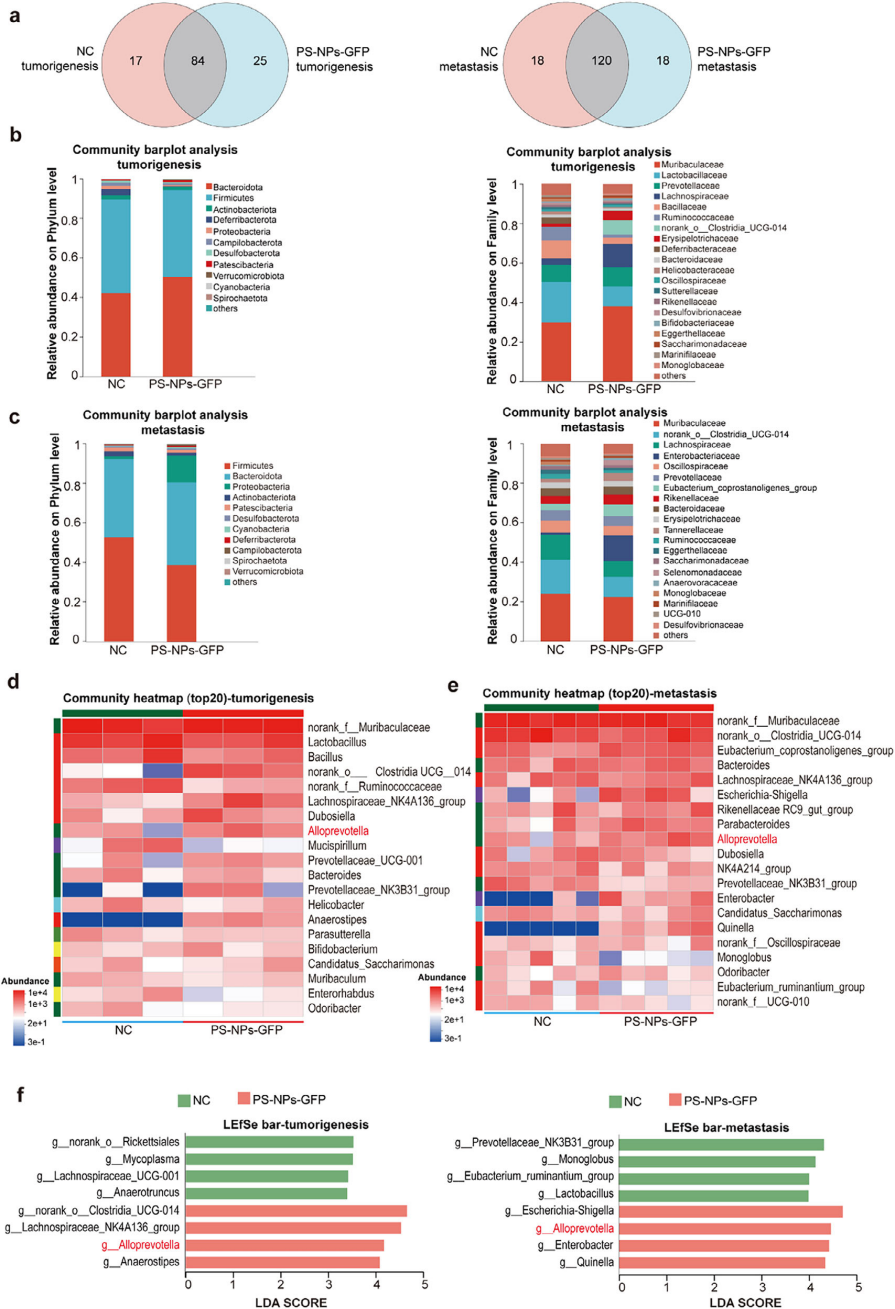
Oral PS‐NPs exposure induced gut microbiota dysbiosis in TNBC‐bearing mice. a), Venn diagram showing group‐specific and shared bacterial genera, with mean relative abundance indicated (tumorigenesis model: n = 3 per group; metastasis model: n = 5 per group). b), Gut microbiota community composition at the phylum and family levels in the tumorigenesis model (n = 3 per group). c), Gut microbiota community composition at the phylum and family levels in the metastasis model (n = 5 per group). d), Heatmap of the top 20 genera identified by MetaStat analysis in the tumorigenesis model. Relative abundance values are Z‐score‐standardized (red, higher; blue, lower) (n = 3 per group). e), Heatmap of the top 20 genera identified by MetaStat analysis in the metastasis model. Relative abundance values are Z‐score‐standardized (red, higher; blue, lower) (n = 5 per group). f), LEfSe analysis showing differentially enriched gut microbial taxa at the genus level between NC and PS‐NPs‐GFP groups. Taxa with a linear discriminant analysis (LDA) score > 3 are shown (tumorigenesis: n = 3 per group; metastasis: n = 5 per group). The linear discriminant analysis (LDA) effect size (LEfSe) for f (http://huttenhower.sph.harvard.edu/LEfSe) was performed to identify the significantly abundant taxa (phylum to genera) of bacteria among the different groups (LDA score > 2, *p* < 0.05).

At the genus level, Venn diagram analysis showed that 126 genera were detected in the tumorigenesis group, with 25 uniquely enriched in PS‐NPs‐treated mice. In the metastasis group, 156 genera were identified, of which 18 were specific to PS‐NPs exposure (**Figure** **3**a). Operational taxonomic unit (OTU)‐based analyses revealed significant alterations in microbial composition and diversity at both phylum and family levels. Compared to the tumorigenesis group, mice in the metastasis group exhibited increased genus‐level microbial abundance. Despite baseline differences between the two models, both shared core microbial features. Firmicutes and Bacteroidota dominated the gut microbiota in all groups, but the Firmicutes/Bacteroidota ratio was markedly reduced following PS‐NPs exposure. In the metastasis group, Proteobacteria abundance was substantially after exposure, a phylum containing multiple pathogenic genera such as *Salmonella*, *Escherichia*, and *Shigella*, commonly recognized as a hallmark of intestinal dysbiosis. At the family level, PS‐NPs exposure decreased Lactobacillaceae and increased Muribaculaceae abundance in the tumorigenesis group (Figure 3b, c).

Heatmap profiling of the top 20 genera revealed consistent microbial shifts across tumorigenesis and metastasis stages. Notably, *Alloprevotella* displayed a robust PS‐NPs‐induced enrichment in both models (Figure 3d, e). Linear discriminant analysis effect size (LEfSe) identified multiple discriminative taxa across taxonomic levels, with *Alloprevotella* emerging as a shared microbial signature linked to PS‐NPs‐driven TNBC progression. In the metastasis group, microbial dysbiosis was more severe, and *Escherichia‐Shigella* showed the highest differential enrichment among potentially pathogenic genera (Figure 3f).

Collectively, these results demonstrate that oral PS‐NPs exposure induces gut microbial dysbiosis characterized by enrichment of *Alloprevotella* and *Escherichia‐Shigella*, implicating microbiota alterations as a potential mediator of TNBC progression and metastasis.

### 2.4 Gut Microbiota‐Derived Glutamic Acid and Platelet Activation Coordinate PS‐NPs‐Induced TNBC Progression

**Figure 4:**
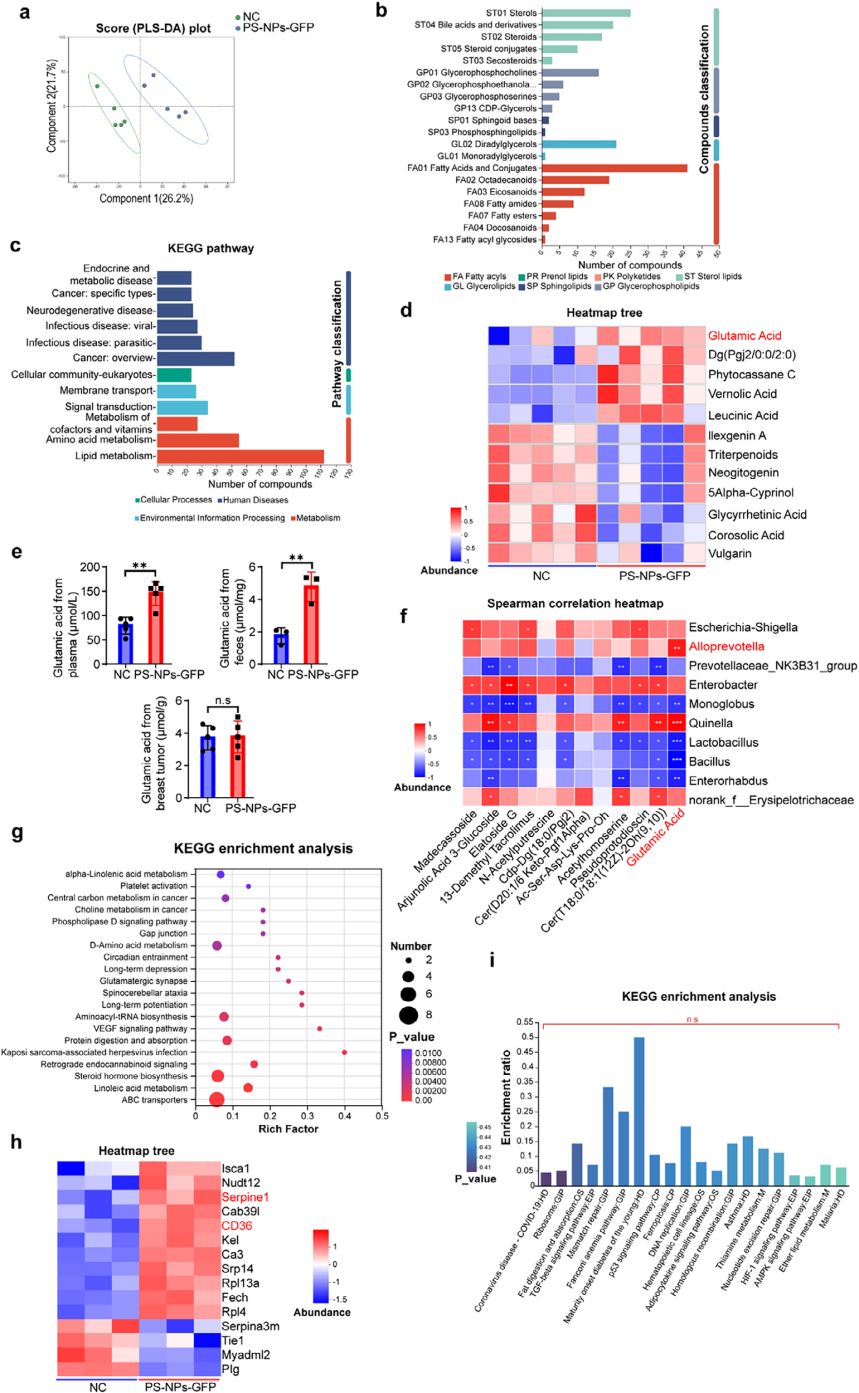
Gut *Alloprevotella*‐derived glutamate and platelet activation coordinated PS‐NPs‐induced TNBC progression. a), Partial least‐squares discriminant analysis (PLS‐DA) score plot of 10 fecal metabolites from NC and PS‐NPs‐GFP groups; each dot represents one mouse (n = 5 per group). b), KEGG pathway enrichment analysis classified by functional categories, highlighting key metabolic processes altered upon PS‐NPs‐GFP exposure (n = 5 per group). c), Differential metabolites identified by untargeted metabolomics and subjected to KEGG pathway enrichment analysis (n = 5 per group). d), Heatmap of the top 12 differential metabolites (excluding those with undefined functions), with relative abundance values Z‐score‐normalized (blue, lower; red, higher) (n = 5 per group). e), Glutamate concentrations in feces (n = 3 per group), serum (n = 5 per group), and breast tumor tissue (n = 5 per group) from NC and PS‐NPs‐GFP mice (mean ± SEM). f), Spearman correlation heatmap between gut microbiota genera and the differential metabolites shown in panel d. The top 10 microbe‐metabolite correlations were displayed (red, positive; blue, negative); blank cells indicate non‐significant associations (n = 5 per group). g), KEGG pathway enrichment analysis of differential metabolites between NC and PS‐NPs‐GFP groups, showing the main perturbed pathways (n = 5 per group). h), Differentially expressed platelet activation‐related proteins between NC and PS‐NPs‐GFP groups (n = 3 per group), with Z‐score‐normalized expression profiles (red, upregulated; blue, downregulated). i, KEGG pathway enrichment of differentially expressed proteins, highlighting major platelet‐associated pathways altered by PS‐NPs exposure (n = 3 per group). Statistical analyses for panels (a–e) and g–i) were performed using unpaired two‐tailed t‐tests. Spearman's correlation in f was considered significant when the correlation coefficient was >0.6 or <−0.6 with *p* < 0.01. Differentially expressed proteins (DEPs) were defined as those with a fold change >1.2 or <0.83 and *p* < 0.05. *p < 0.05; **p < 0.01; ***p < 0.001; n.s., not significant.

Gut microbiota modulate host physiology partly through the production of bioactive metabolites that enter systemic circulation after intestinal absorption. To examine whether PS‐NPs‐induced dysbiosis contributes to TNBC progression, untargeted metabolomic profiling of fecal samples from advanced metastatic TNBC mice was performed using laser capture microdissection (LCM) and gas chromatography‐mass spectrometry (GC‐MS). Partial least squares discriminant analysis (PLS‐DA) revealed distinct metabolic signatures between PS‐NPs‐exposed and control mice (**Figure** **4**a). KEGG annotation identified fatty acids and conjugates as the dominant metabolite class (Figure 4b), with lipid metabolism identified as the most significantly enriched pathway (Figure 4c).

Among differentially abundant metabolites, glutamic acid showed the greatest upregulation in PS‐NPs‐treated mice (Figure 4d). As a precursor of α‐ketoglutarate, glutamic acid is central to lipid metabolism and implicated in cancer‐associated metabolic reprogramming, potentially enhancing proliferative, invasive, and immunomodulatory processes via autocrine/paracrine signaling.^[^ ^21^ ^]^ Elevated glutamic acid was detected in both feces and plasma, but not in orthotopic tumors, suggesting a predominantly gut microbiota‐derived origin (Figure 4e). Spearman correlation analysis integrating microbiome and metabolome datasets revealed a strong positive association between glutamic acid and *Alloprevotella* abundance (Figure 4f), implicating this taxon as a potential metabolite source. KEGG analysis further identified perturbations in multiple lipid‐related pathways, including choline metabolism, steroid hormone biosynthesis, and α‐linolenic/linoleic acid metabolism, as well as enrichment of the platelet activation pathway (Figure 4 g).

Given prior observations of reduced platelet counts and increased microthrombus formation in metastatic sites, platelet involvement was investigated. Proteomic profiling of platelets from late‐stage TNBC mice demonstrated upregulation of CD36 and Serpine1 in PS‐NPs‐treated animals (Figure 4 h). CD36, a lipid transporter and platelet signaling activator, promotes TNBC metastasis,^[^ ^22^, ^23^ ^]^ while Serpine1, a fibrinolysis inhibitor, is linked to platelet hyperactivation and poor breast cancer prognosis.^[^ ^24^, ^25^ ^]^ KEGG analysis revealed modulation of the TGF‐β, p53, HIF‐1, and AMPK signaling pathways (Figure 4i). Impaired AMPK pathway, a central regulator of lipid metabolism, is known to enhance platelet hyperreactivity via downstream HIF‐1α and NF‐κB/p65 signaling, leading to transcriptional upregulation of CD36.^[^ ^26^, ^27^, ^28^, ^29^ ^]^

To mechanistically validate the glutamate‐platelet axis in PS‐NPs‐driven TNBC, pharmacological inhibition were applied. Treatment with the glutamate receptor antagonist memantine hydrochloride (C_12_H_21_N·HCl) or the CD36 inhibitor sulfosuccinimidyl oleate sodium (SSO) markedly reduced PS‐NPs‐induced pulmonary metastases and attenuated platelet activation (Figure S5a–c, Supporting Information). Both inhibitors also restored AMPK signaling in platelets and normalized CD36 and Serpine1 expression levels (Figure S5d, Supporting Information). In the *MMTV‐PyMT* transgenic model, C_12_H_21_N·HCl suppressed PS‐NPs‐driven mammary tumorigenesis, reduced CD42b^+^ platelet thrombus formation in primary tumors, and mitigated peripheral platelet activation (Figure S6a–d, Supporting Information).

Overall, these findings identify glutamic acid as a key *Alloprevotella*‐derived metabolite driving PS‐NPs‐induced TNBC progression via platelet activation.

### 2.5 Oral PS‐NPs Exposure Promotes TNBC Initiation and Progression via Stemness Induction, EMT Activation, and Lipid Metabolic Reprogramming

To elucidate the mechanisms by which oral PS‐NPs accelerate TNBC development, we examined systemic alterations involving gut microbiota dysbiosis, lipid metabolic reprogramming, and platelet activation‐pathways identified as critical effectors in advanced disease stage. To uncover stage‐specific molecular drivers, comparative proteomic profiling was performed on early‐ and late‐stage TNBC tissues following PS‐NPs ingestion.

**Figure 5:**
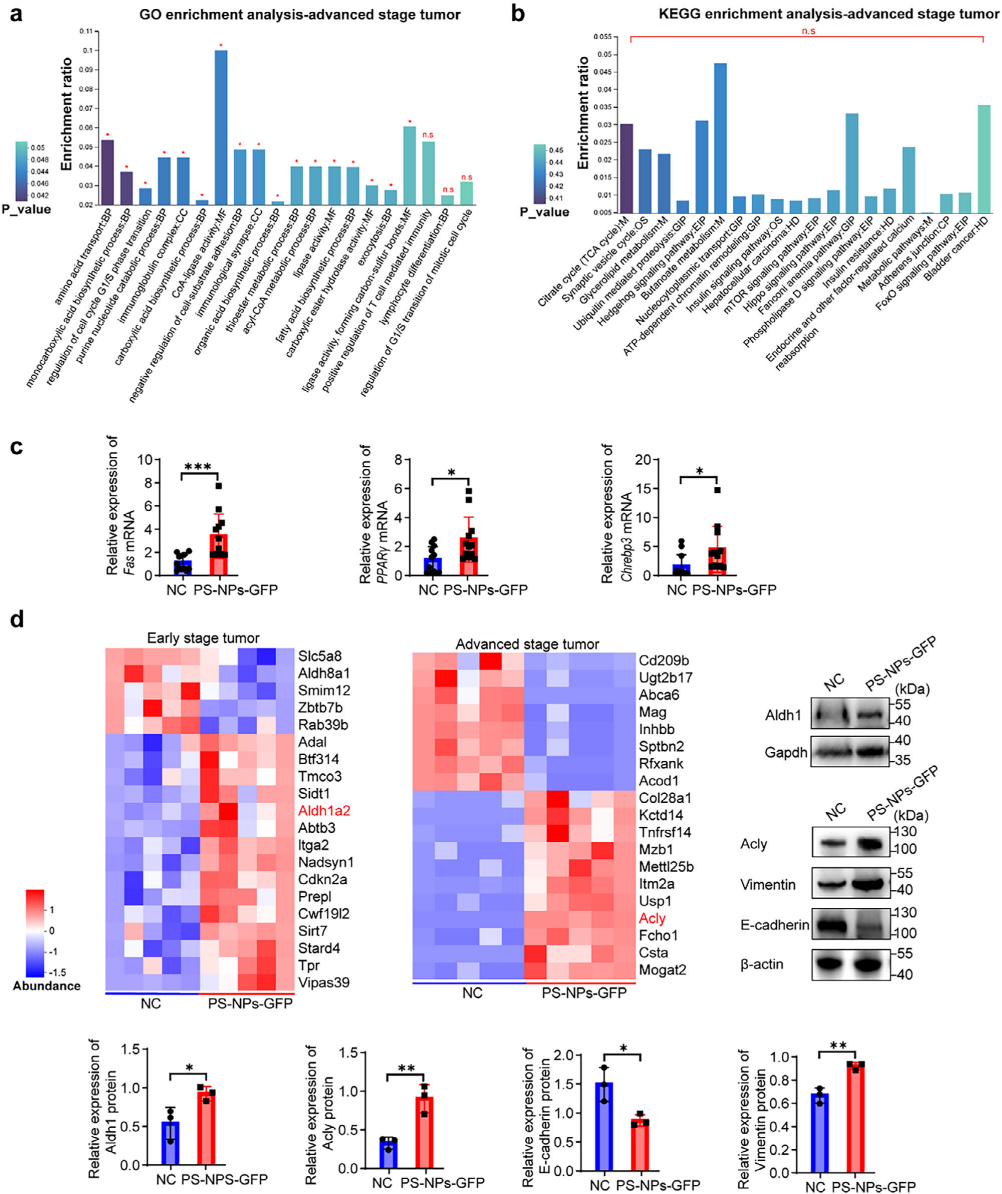
Proteomic analysis revealed PS‐NPs‐induced dysregulation of lipid metabolism, stemness, and epithelial‐mesenchymal transition in TNBC. a), Gene Ontology (GO) enrichment analysis of the top 50 differentially expressed proteins in advanced‐stage TNBC, categorized biological process (BP), molecular function (MF), and cellular component (CC) (n = 5 per group). b), KEGG pathway enrichment of the top 50 differentially expressed proteins, highlighting key pathways altered by PS‐NPs exposure in advanced‐stage TNBC (n = 5 per group). c), Relative mRNA expression of *Fas*, *PPARγ*, *Chrebp3* in advanced‐stage tumors from NC and PS‐NPs‐GFP groups measured by qRT‐PCR. Expression normalized to *Gapdh* and calculated via the 2^–ΔΔCt^ method (n = 3 per group; mean ± SEM). d), Heatmap of differentially expressed proteins associated with tumorigenesis pathways between NC and PS‐NPs‐GFP groups (Z‐score normalized; red, upregulated; blue, downregulated; n = 5 per group). Western blot validation of Aldh1, Acly, E‐cadherin, and Vimentin protein levels with Gapdh and β‐actin as loading controls (n = 3 per group). Quantification was performed using Image J and normalized to internal controls (mean ± SEM). Statistical significance was determined by unpaired two‐tailed t‐tests for panels a‐d; DEPs were defined by fold change >1.2 or <0.83 and P_value < 0.05. *p < 0.05; **p < 0.01; ***p < 0.001.

In early‐stage tumors, PS‐NPs exposure did not significantly alter canonical metabolic pathways (Figure S7a,b, Supporting Information). In contrast, late‐stage tumors exhibited pronounced metabolic remodeling. Gene Ontology (GO) enrichment of the top 50 differentially expressed proteins highlighted upregulation of lipase activity, amino acid transport, and fatty acid biosynthetic process, with CoA‐ligase activity most prominently enriched (**Figure** **5**a). As a central intermediate in lipid homeostasis, acyl‐CoA supports both fatty acid synthesis and β‐oxidation, enabling membrane biogenesis and energy production in rapidly proliferating cancer cells.^[^ ^30^ ^]^ KEGG analysis further identified enrichment in the citrate cycle (TCA cycle), glycerolipid metabolism, and butanoate metabolism (Figure 5b), consistent with augmented glycolysis, fatty acid oxidation, and lipogenesis. In line with these findings, lipid metabolism‐associated genes (*Fas*, *PPARγ*, and *Chrebp3*) were markedly upregulated in late‐stage tumors (Figure 5c), indicating a metabolic shift toward anabolic lipid synthesis.

Proteomic profiling also revealed stage‐dependent molecular signatures beyond metabolism. In early‐stage tumors, the stem cell marker aldehyde dehydrogenase 1 (Aldh1) was significantly upregulated following PS‐NPs exposure. Elevated Aldh1 is linked to enhanced self‐renewal capacity, proliferative potential, and oncogenic susceptibility through genetic and epigenetic reprogramming, and may facilitate immune evasion by suppressing CD8⁺ T cell function,^[^ ^25^, ^31^ ^]^ suggesting that PS‐NPs promote tumor initiation via stemness induction. In late‐stage tumors, Acly, a key metabolic enzyme linking glucose catabolism to lipid biosynthesis by generating acetyl‐CoA from citrate, was markedly elevated. Acly has been implicated in tumor progression across multiple cancer types.^[^ ^32^ ^]^ Consistently, western blot analysis demonstrated increased vimentin and decreased E‐cadherin expression, indicating PS‐NPs‐induced EMT (Figure 5d).

Mechanistic intervention studies further supported these observations: pharmacological inhibition of glutamate receptors with C_12_H_21_N·HCl attenuated PS‐NPs‐induced Aldh1 upregulation in early‐stage tumors, as well as Acly overexpression and EMT marker alterations in late‐stage tumors (Figure S7c, Supporting Information).

Taken together, these findings demonstrate that oral PS‐NPs exposure facilitates TNBC initiation by enhancing stem‐like traits and drives tumor progression via EMT activation. Both processes are underpinned by lipid metabolic reprogramming, with Acly‐mediated lipogenesis emerging as a central node in PS‐NPs‐induced malignancy.

### 2.6 *Alloprevotella* as a Microbial Mediator of TNBC Initiation and Progression via Glutamate Production and Platelet Activation

*Alloprevotella*, a genus within the Prevotellaceae family, has been implicated in metabolic disorders, including diabetes and several cancer types.^[^ ^33^, ^34^ ^]^ In this study, oral PS‐NPs exposure led to marked enrichment of *Alloprevotella* in the gut microbiota, consistently emerging as a microbial signature associated with both TNBC initiation and metastasis.

**Figure 6:**
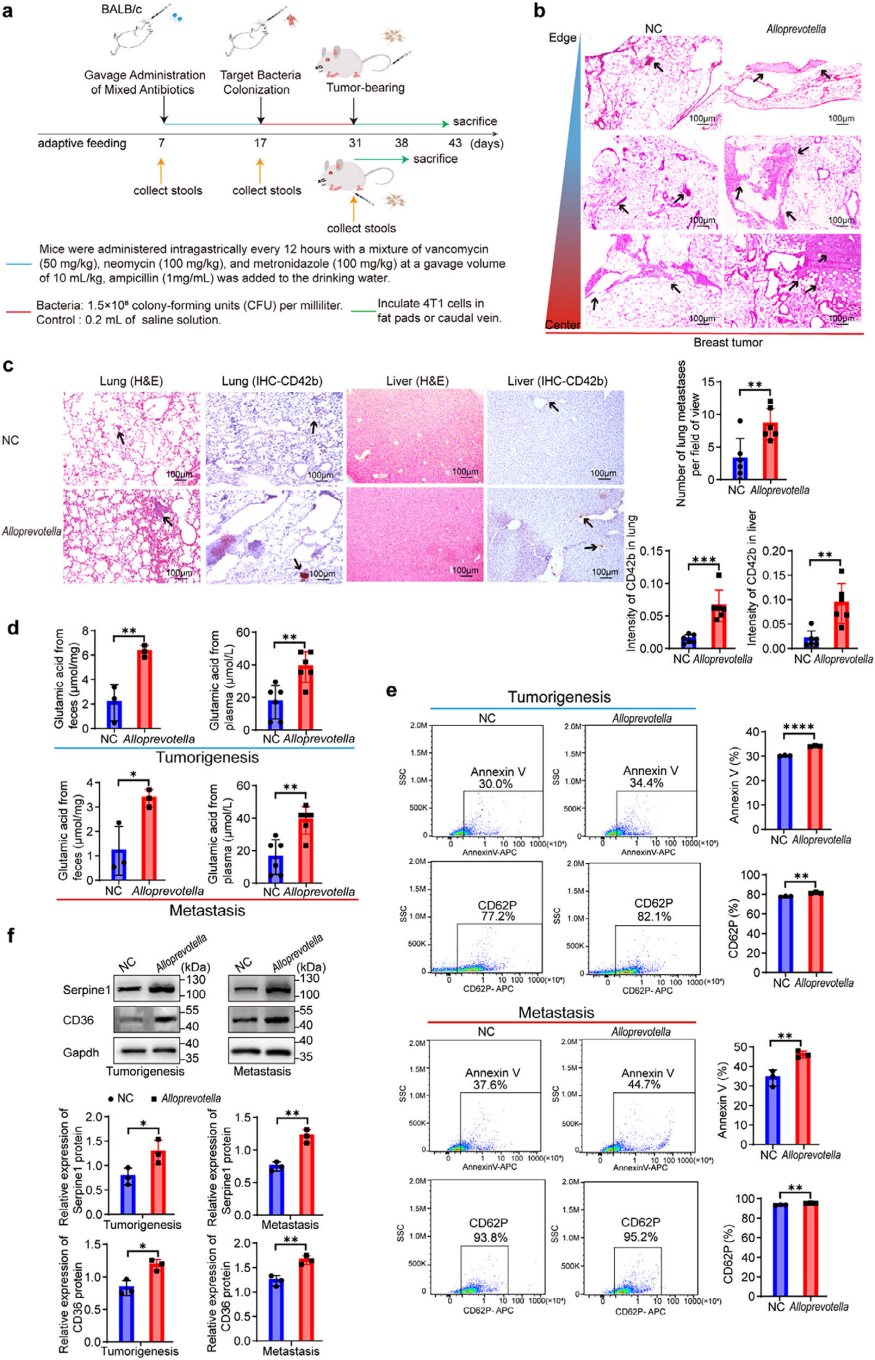
*Alloprevotella* promoted breast cancer initiation and metastasis via glutamate production and platelet activation. a), Experimental design. Seven‐week‐old BALB/c mice received a 10‐day oral gavage of broad‐spectrum antibiotics to deplete gut microbiota, followed by 14 days of targeted microbiota transplantation with donor feces. Subsequently, mice were injected with 4T1 cells orthotopically (tumorigenesis cohort) or via the tail vein (metastasis cohort). Mice were sacrificed 7 days (tumorigenesis) or 12 days (metastasis) post‐injection (n = 6 per group). b), Representative H&E‐stained smammary tumor sections at endpoint (n = 6 per group). c), Quantification of lung metastatic foci by H&E staining. Representative IHC images of lung and liver tissues stained for CD42b with corresponding quantification of staining scores (mean ± SEM; n = 6 mice per group). d), Glutamate concentrations measured in feces (n = 3 per group) and serum (n = 6 per group) from NC and *Alloprevotella*‐colonized mice in both tumorigenesis and metastasis models (mean ± SEM). e), Flow cytometry analysis of platelet activation. Representative plots of Annexin V and CD62P expression on platelets isolated from NC and *Alloprevotella* groups (mean ± SEM; n = 3 per group). Platelets were gated by forward and side scatter and stained with fluorochrome‐conjugated antibodies. Percentages of positive cells were indicated. Data represented at least three independent experiments. f), Western blot analysis of CD36 and Serpine1 expression in platelets from NC and *Alloprevotella* groups (n = 3 per group). Gapdh served as loading control. Representative immunoblots and densitometric quantification normalized to Gapdh were shown (mean ± SEM). Statistical analysis was performed using unpaired two‐tailed t‐tests for panels (c–f). *p < 0.05; **p < 0.01; ***p < 0.001; ****p < 0.0001.

To investigate its functional role, pseudo‐germ‐free mice were generated via a 10‐day broad‐spectrum antibiotic regimen, resulting in stable gut microbiota depletion. These mice were subsequently gavaged with *Alloprevotella* for 14 days, achieving successful intestinal colonization. TNBC initiation and metastatic progression were modeled by orthotopic or intravenous injection of 4T1 cells, respectively (**Figure** **6**a; Figure S8a–c, Supporting Information). *Alloprevotella* colonization significantly enhanced primary tumorigenesis and lung metastasis (Figure 6b,c). Concurrently, glutamate levels were markedly elevated in both feces and serum (Figure 6d), implicating *Alloprevotella*‐derived glutamate in establishing a tumor‐promoting circulatory metabolic network.

Consistent with increased metastatic burden, immunohistochemistry (IHC) analyses revealed abundant platelet‐rich thrombi in the lung and liver (Figure 6c). Flow cytometry demonstrated elevated expression of platelet activation markers Annexin V and CD62P in colonized mice (Figure 6e). Western blot analyses further confirmed increased levels of CD36 and Serpine1 (Figure 6f), suggesting enhanced platelet activation and potential involvement in lipid metabolic crosstalk during tumor progression.

In summary, these results identify *Alloprevotella* as a critical gut microbial driver in PS‐NPs‐promoted TNBC, acting through glutamate production and platelet activation to facilitate tumor initiation and metastatic dissemination.

### 2.7 Glutamate and Activated Platelet Synergistically Enhance Migration, Invasion, and Stemness of TNBC Cells

To delineate the contributions of glutamate and platelets to TNBC malignant phenotypes, we conducted in vitro assays using human (MDA‐MB‐231, HCC1937, BT‐549) and murine (4T1) TNBC cell lines. MDA‐MB‐231 and BT‐549 represent mesenchymal‐like subtypes, HCC1937 belongs to the basal‐like 1 subtype, and 4T1 is also mesenchymal‐like.

**Figure 7:**
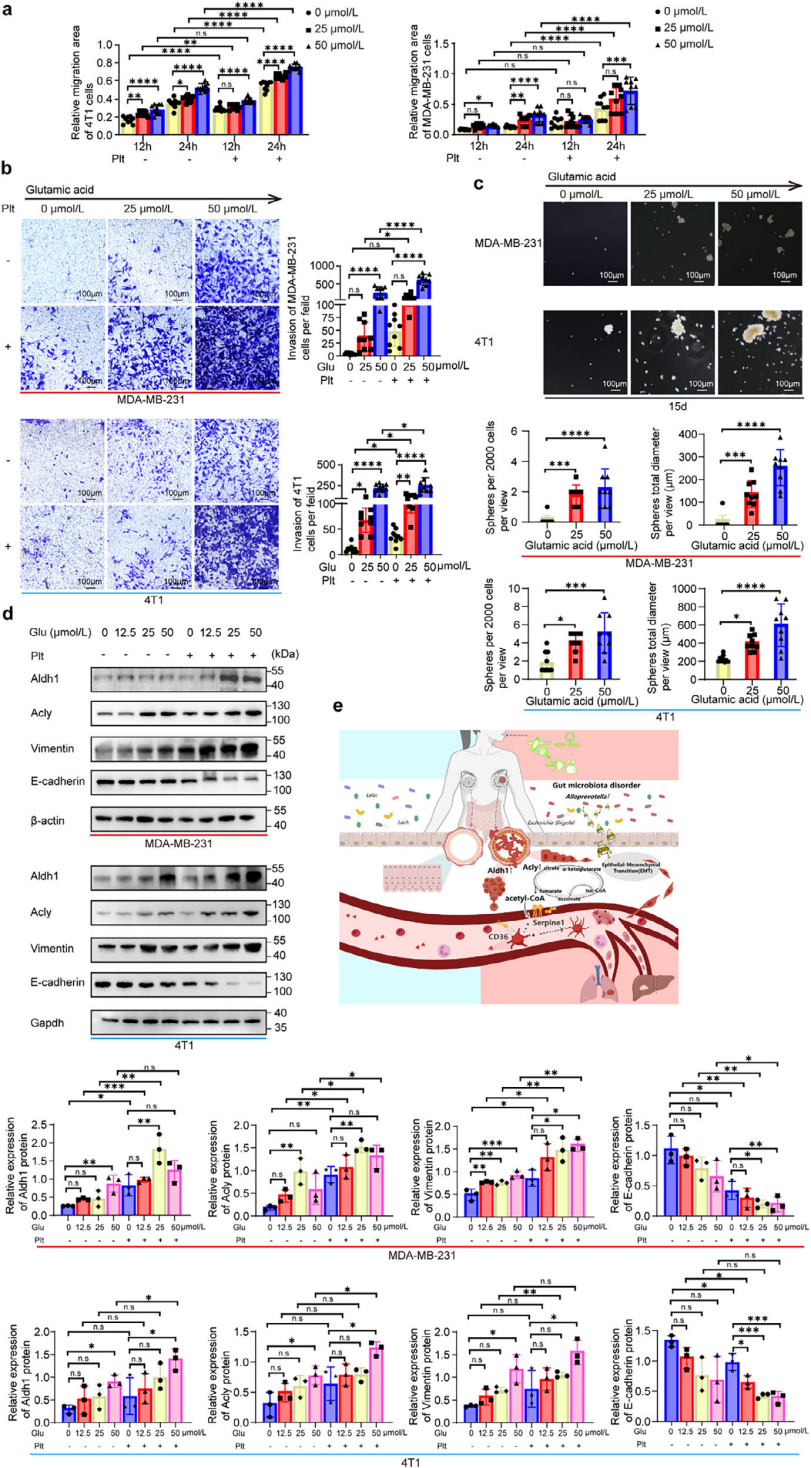
Glutamate and platelet stimulation enhanced migration, invasion, and stem‐like phenotypes in TNBC cells. a), Wound healing assays assessing migration of 4T1 and MDA‐MB‐231 cells treated with increasing concentrations of glutamate (0, 25, 50 µmol L^−1^) and/or platelets. Wound closure was quantified using Image J and presented as mean ± SEM (n = 3 independent experiments). b), Transwell assays of MDA‐MB‐231 and 4T1 cells treated with glutamate (0, 25, 50 µmol L^−1^) and/or platelets. Invaded cells were stained with crystal violet and quantified in five random fields per well. Data were mean ± SEM (n = 3 independent experiments). c), Quantification of number and diameter of tumor spheroids (>50 µm) formed by 4T1 and MDA‐MB‐231 cells treated with glutamate (0, 25, 50 µmol L^−1^) (n = 3 independent experiments). d), Western blot analysis of Acly, Aldh1, EMT markers, and loading controls (Gapdh or β‐actin) in 4T1 and MDA‐MB‐231 cells treated with glutamate (0, 12.5, 25, 50 µmol L^−1^) with or without platelet co‐incubation for 24 h. Representative immunoblots and densitometric quantification normalized to loading controls were shown (mean ± SEM; n = 3 independent experiments). e), Schematic illustrating the proposed mechanism by which *Alloprevotella*‐derived glutamate and platelet activation synergistically promote TNBC tumorigenesis and metastasis following oral PS‐NPs exposure. Statistical analysis was performed using one‐way ANOVA with Dunnett's post hoc test and unpaired two‐tailed t‐tests for panels a, b, and d; and one‐way ANOVA with Dunnett's post hoc test for panels c. *p < 0.05; **p < 0.01; ***p < 0.001; ****p < 0.0001; n.s, not significant.

Wound healing and transwell assays demonstrated a dose‐dependent increase in migration and invasion following glutamate treatment, with maximal effects at 50 µmol L^−1^. Platelet co‐stimulation further potentiated these effects, whereas platelets alone elicited a moderate pro‐migratory and pro‐invasive response (**Figure** **7**a,b; Figures S9a and S10a,b, Supporting Information). Consistently, glutamate significantly increased tumorsphere number and cumulative diameter, indicating enhanced cancer stemness (Figure 7c; Figure S10c, Supporting Information). Due to the limited lifespan of platelets in vitro, their effects on sphere formation were not assessed.

In vivo, the pro‐metastatic effect of PS‐NPs was evident across TNBC subtypes. In metastatic models using 4T1 cells and Py8119 cells (a basal‐like/immunomodulatory hybrid subtype), PS‐NPs exposure increased metastatic burden in both cell types (Figure 2a–h; Figure S3b,c, Supporting Information).

Mechanistically, western blot analysis revealed that glutamate upregulated Acly, Aldh1, and EMT‐related proteins in a concentration‐dependent manner. Co‐treatment with platelets further amplified these effects, whereas platelet stimulation alone induced a similar but less pronounced changes (Figure 7d).

Altogether, these findings indicate that PS‐NPs dietary exposure promotes *Alloprevotella*‐derived glutamate production, and elevated systemic glutamate levels, together with activated platelets, synergistically drive TNBC initiation and hematogenous metastasis. This effect is likely mediated through enhanced stemness, Acly‐driven lipid metabolic reprogramming, and EMT activation, highlighting a coordinated pro‐tumorigenic axis linking gut microbiota, metabolism, and platelet activation (Figure 7e).

## 3 Discussion

TNBC is marked by pronounced heterogeneity, aggressive invasiveness, and poor clinical prognosis. Its etiology involves a multifaceted interplay between intrinsic determinants, including genetic, physiological, and immunological factors, and extrinsic influences such as environmental pollutants, lifestyle, and socioeconomic status. Emerging evidence increasingly implicates environmental exposures in TNBC pathogenesis. For instance, large multiethnic cohort studies have linked ambient PM2.5 exposure to elevated breast cancer risk,^[^ ^35^ ^]^ while chronic low‐dose exposure to endocrine‐disrupting chemicals (EDCs) like bisphenol A and phthalates, common in consumer products, has been shown to promote mammary tumorigenesis.^[^ ^36^ ^]^ NPs, as novel environmental contaminants, can enter the body via inhalation or ingestion, potentially triggering chronic inflammation, genomic instability, and lipid metabolic dysregulation‐key hallmarks of carcinogenesis.^[^ ^37^, ^38^ ^]^ Despite this, their role in TNBC development remains poorly understood.

In this study, we systematically investigated the impact of oral PS‐NPs exposure on TNBC initiation and progression across multiple in vivo models. Our data demonstrate that PS‐NPs significantly enhance tumor initiation and metastatic dissemination, while exerting limited influence on primary tumor growth. Unlike prior studies that primarily focused on canonical oncogenic drivers, we uncover a distinct mechanism whereby PS‐NPs potentiate tumor initiation through increased cancer stemness and facilitate metastasis via Acly‐dependent fatty acid metabolism and EMT activation.

Mechanistically, integrated multi‐omics analyses, supported by in vivo and in vitro validation, identify the gut microbiota, its metabolites, and platelets as central mediators of PS‐NPs‐induced TNBC progression. The genus *Alloprevotella* and its metabolite glutamate emerged as key contributors. Among blood components, platelets exhibited the most pronounced response to PS‐NPs exposure, playing a pivotal role in metastasis promotion. Activated platelets likely enhance lipid metabolic crosstalk with tumor cells and release pro‐metastatic soluble factors, collectively fostering TNBC malignancy. This cascade appears driven by PS‐NPs‐induced systemic metabolic reprogramming and platelet modulation by gut‐derived metabolites. In vitro assays confirm a synergistic interaction between glutamate and platelets that amplified TNBC cell migration and invasion. Together, these findings reveal a complex gut microbiota‐glutamate‐platelet axis underlying TNBC progression, although detailed molecular mechanisms warrant further elucidation.

Glutamate, beyond its classical neurotransmitter function, acts as a critical metabolic substrate supporting the anabolic demands of rapidly proliferating cancer cells. Mutations in metabolic genes can enhance glutamate dependency in diverse cancers, and glutamylation of microtubules has been implicated in breast cancer metastasis.^[^ ^39^, ^40^ ^]^ Consistent with this, we observed elevated glutamate levels in fecal and plasma samples following PS‐NPs exposure, with *Alloprevotella* identified as a predominant microbial source. While endogenous synthesis and alternative microbial pathways may contribute, circulating glutamate likely functions as a systemic metabolic modulator that promotes TNBC progression via platelet activation and downstream oncogenic signaling. The role of tumor‐intrinsic glutamate production remains an important topic for future research.

Notably, *Shigella* was markedly enriched after PS‐NPs exposure and promoted TNBC lung metastasis independently of glutamate or platelet activation (Figure 3f; Figures S8c and S11a–d, Supporting Information). Intriguingly, *Shigella* treatment increased CD3 and PD‐1 expression within the metastatic lung niche and elevated PD‐1 in the liver, indicative of T cell infiltration and an “immune‐inflamed” tumor phenotype. Western blot analyses confirmed MAPK pathway activation in primary tumors, yet systemic inflammatory cytokines (IL‐6, IL‐10, MCP‐1, IFN‐γ, TNF‐α, IL‐12) remained unaltered (Figure S11e–g, Supporting Information), suggesting *Shigella*’s pro‐tumorigenic effects are driven by localized inflammation rather than broad systemic inflammation. This delineates a mechanistically distinct microbial‐metabolic‐hematologic axis from the *Alloprevotella*‐glutamate‐platelet pathway, underscoring the specificity of microbial crosstalk in TNBC progression.

We acknowledge several limitations that warrant further investigation. Toxicological outcomes are strongly influenced by nanoparticle size, surface properties, and exposure routes.^[^ ^41^ ^]^ In this study, we focused on orally administered PS‐NPs, a commonly detected species, to explore indirect carcinogenic mechanisms mediated by gut microbiota and systemic responses. Although direct pro‐carcinogenic effects of MPs within tumors have been reported, current analytical constraints hinder reliable detection‐particularly due to particle degradation during sample processing and the difficulty in recovering MPs of the specific size used in our study. As a result, measured MP levels in tumors were even lower than the environmental background. Nevertheless, our findings underscore that the possibility of in vivo accumulation of environmentally derived MPs should not be overlooked, and the long‐term biological consequences of chronic exposure remain to be fully elucidated. Moreover, smaller PS‐NPs capable of translocating across the intestinal barrier into the circulation may exert direct effects on distal organs, potentially contributing to TNBC progression through alternative pathways.

In summary, this work reveals a previously unrecognized mechanism whereby oral PS‐NPs exposure accelerates TNBC progression through synergistic modulation of gut microbiota‐derived glutamate and platelet activation (Figure 7e). The dynamic interplay among microbial metabolites, platelet activity, and tumor cell behavior enhances our understanding of environmental pollutant‐driven tumorigenesis and provides a theoretical foundation for developing innovative multi‐targeted interventions to mitigate pollution‐associated cancer risk.

## 4 Experimental Section

### Animals

All animal experiments were approved by the Laboratory Animal Research Center of Zhejiang Chinese Medical University (Approval No. [IACUC‐202504‐25]). Female BALB/c mice (6‐8 weeks old) were purchased from Hangzhou Qizhen Laboratory Animal Technology Co., Ltd. Transgenic *MMTV‐PyMT* mice^[^ ^42^ ^]^ were generously provided by Prof. Junling Liu (Shanghai Jiao Tong University School of Medicine) and maintained on a C57BL/6J background by in‐house breeding. Ten‐week‐old *MMTV‐PyMT* mice bearing spontaneous mammary tumors were used for tumor studies, with a minimum of five mice per group. Primer sequences are listed in Table S1 (Supporting Information). All mice were housed under specific pathogen‐free (SPF) conditions with a 12 h light/dark cycle, 50 ± 5% relative humidity, and a temperature of 22 ± 2 °C. Experimental and control groups maintained in separate cages to prevent cross‐contamination.

### Cell Culture

The human TNBC cell lines MDA‐MB‐231 (RRID: CVCL_0062; ATCC HTB‐26), HCC1937 (RRID: CVCL_0290; ATCC CRL‐2336), and BT‐549 (RRID: CVCL_1092; ATCC HTB‐122), as well as the murine TNBC cell lines 4T1 (RRID: CVCL_0125; ATCC CRL‐2539) and Py8119 (RRID: CVCL_AQ09), were obtained from the American Type Culture Collection (ATCC). MDA‐MB‐231 and 4T1 cells were maintained in Dulbecco's Modified Eagle Medium (DMEM) supplemented with 10% fetal bovine serum (FBS; Gibco, B210695RP) and 1% penicillin‐streptomycin (P/S; Biosharp, BL505A). HCC1937 and BT‐549 cells were maintained in RPMI‐1640 medium supplemented with 10% FBS and 1% P/S. Py8119 cells were maintained in Ham's F‐12 nutrient medium (F12) supplemented with 5 µg mL^−1^ insulin (Yeasen, 40112ES25), 1 µg mL^−1^ hydrocortisone (Yeasen, 40109ES08), 10 ng mL^−1^ epidermal growth factor (Yeasen, 92701ES60), 10% FBS, 1% P/S, and 50 µg mL^−1^ gentamycin sulfate salt (Yeasen, 60214ES). All cells were cultured at 37 °C in a humidified atmosphere with 5% CO_2_, routinely authenticated, and confirmed to be mycoplasma‐free.

### In vivo Breast Cancer Colonization

Mice were anesthetized with isoflurane (5 µL g^−1^ body weight) using a precision vaporizer prior to cell implantation. For metastatic colonization assays, 5 × 10^5^ 4T1 cells were injected into the tail vein. For orthotopic tumor models, 2 × 10^6^ 4T1 or Py8119 cells were inoculated into the mammary fat pad of BALB/c mice. Surgical sites were sutured post‐injection to ensure wound closure.

### PS‐NPs and in Vivo Tracking

Commercial monodisperse green fluorescent PS‐NPs (100 nm; FF3301G, Rigor) labeled with GFP were characterized by visual inspection and SEM (Hitachi SU8010) prior to use. For biodistribution analysis, tissues were collected after PS‐NPs exposure, embedded in OCT compound, and snap‐frozen at −80 °C. Cryosections (5 µm) were prepared using a cryostat (CryoStar NX50, Epredia), and fluorescence signals were visualized with an inverted fluorescence microscope (ECLIPSE Ts2R‐FL, Nikon).

### Germ‐Free Mouse Construction

An antibiotic cocktail containing vancomycin (50 mg kg^−1^), metronidazole (100 mg kg^−1^), and neomycin (100 mg kg^−1^) (all from Shanghai Yuanye Bio‐Technology) was administered via oral gavage (10 µL g^−1^ body weight) every 12 h. Drinking water was supplemented with ampicillin (1 mg mL^−1^, Shanghai Yuanye Bio‐Technology) ad libitum. After 10 days of treatment, fecal samples were collected for DNA extraction and microbiome analysis.

### Bacterial Culture and Colonization

*Alloprevotella rava* (BNCC 241112) was cultured anaerobically at 37 °C on Columbia blood agar plates. *Shigella dysenteriae* (CGMCC 1.1869) was obtained from the China General Microbiological Culture Collection Center and cultured aerobically at 37 °C on standard agar medium. For colonization experiments, bacterial colonies were harvested, resuspended in sterile saline (10^8^ CFU/mL), and administered via oral gavage (10 µL g^−1^ body weight) every other day for 14 days.

### H&E Staining and Immunohistochemistry

Mouse organs were fixed in 4% paraformaldehyde for 36 h, dehydrated through a graded ethanol series, and embedded in paraffin. Sections (5 µm) were prepared for H&E staining and IHC analysis. For IHC, antigen retrieval was performed by immersing the sections in 0.1 m sodium citrate buffer and heating until the solution reached boiling point, followed by incubation in a 95 °C water bath for 15 min and natural cooling to room temperature. Endogenous peroxidase activity was blocked by incubation with 3% hydrogen peroxide for 10 min, followed by thorough rinsing with distilled water. Non‐specific binding was blocked by incubating the sections with 10% goat serum (Beyotime Biotechnology, C0265) at 37 °C for 2 h. The sections were then incubated overnight with anti‐CD42b antibody (Abcam, ab183345, 1:200) at 4 °C. After rinsing with distilled water, biotin‐labeled goat anti‐rabbit IgG (Beyotime Biotechnology, A0277, 1:1000) was applied at 37 °C for 30 min. Immunoreactivity was visualized using a DAB detection kit, and sections were counterstained with hematoxylin. Positive staining of CD42b was quantitatively analyzed using Image J software.

### Hematological Analysis

Peripheral blood (50 µL) was collected via orbital puncture into EDTA‐coated tubes and diluted 1:3 in sterile saline. Hematological parameters were measured using an automated hematology analyzer (XN‐1000V‐B1, Sysmex).

### RNA Extraction and RT‐qPCR

Tissues (50 mg) were homogenized in Freezol Reagent (Vazyme) supplemented with dilution buffer (Vazyme) at a 5:1 ratio. Following vortexing and 5 min incubation, samples were centrifuged at 12000 rpm for 5 min. The supernatant was mixed with isopropanol, incubated for 5 min, and centrifuged at 12000 rpm for 10 min. The resulting pellet was washed twice with 75% ethanol, centrifuged at 8000 g for 3 min, air‐dried, and dissolved in ddH_2_O. RNA concentration and purity were determined using a NanoDrop spectrophotometer (Thermo Scientific).

For cDNA synthesis, 1 µg of RNA was reverse transcribed using 4×gDNA Wiper Mix (Vazyme) and 5×Hiscript Mix (Vazyme) in a 20 µL reaction volume. RT‐qPCR was performed on a Real‐Time PCR Analyzer (Big Fish) using ChamQ SYBR qPCR Master Mix (Vazyme). The thermal cycling protocol was: 95 °C for 30 s; 45 cycles of 95 °C for 5 s and 55 °C for 30 s; followed by melting curve analysis (95 °C for 5 s, 60 °C for 1 min, 95 °C for 15 s). Data were analyzed using QuantFinder 96 Professional software (Big Fish). Primer sequences are provided in Table S1 (Supporting Information) (synthesized by Shanghai Sangon Biotech).

### Western Blotting

Fresh mouse tissues (50 mg) were homogenized in 1×RIPA buffer (Beyotime Biotechnology) containing 1% PMSF (Beyotime Biotechnology) and centrifuged at 12000 rpm for 20 min at 4 °C. Protein concentrations were determined using a BCA assay. Equal amounts of protein (50 µg) were resolved by 10% SDS‐PAGE and transferred onto PVDF membranes (0.22 µm; Vazyme). Membranes were blocked with 5% skim milk for 2 h at room temperature, followed by overnight incubation at 4 °C with the following primary antibodies: anti‐Serpine1 (Cohesion, CPA6014, 1:500), anti‐E‐cadherin (Proteintech, 20874‐1‐AP, 1:1000), anti‐Vimentin (Abcam, EPR3776, 1:1000), anti‐Aldh1 (Cohesion, CPA3760, 1:500), anti‐CD36 (Cohesion, CQA1879, 1:500), anti‐Acly (Proteintech, 15421‐1‐AP, 1:1000), ERK1/2 (HUABIO, SA43‐03, 1:5000), phospho‐ERK1 (T202+Y204)/ERK2 (T185+Y187) (HUABIO, SC58‐01, 1:5000), p38α/MAPK14 (HUABIO, JF55‐07, 1:2000), phospho‐p38 (T180+Y182) (HUABIO, ER2001‐52, 1:2000), JNK1/2/3 (HUABIO, SA43‐06, 1:2000), AMPK1 (Epizyme, 19B13C41, 1:1000), phospho‐AMPKα1 (Thr183)/AMPKα2 (Thr172) (Epizyme, 48F92M22, 1:1000), βII‐tubulin (HUABIO, ST52‐04, 1:5000), β‐actin (Yeasen, 30102ES60, 1:10000), and Gapdh (HUABIO, EMI1901‐57, 1:5000). After washing, membranes were incubated for 2 h at room temperature with HRP‐conjugated goat anti‐mouse IgG (Absin, abs20039ss, 1:5000) or goat anti‐rabbit IgG (Abcam, ab205718, 1:4000). Protein bands were visualized using a Tanon 5200 Multi imaging system and quantified with Image J software.

### Flow Cytometric Analysis of Platelet CD62P and Annexin V Expression

Blood was collected from mice into sodium citrate and supplemented with apyrase (Sigma, A6535; 1:2000) and PGE1 (Sigma, 900100P; 1:10000) to prevent platelet activation. Platelet‐rich plasma was obtained by centrifugation at 230 × g for 10 min, supplemented with apyrase and EDTA (1:100), and centrifuged at 800 × g for 10 min. The resulting platelet pellets were resuspended in Tyrode's buffer to generate washed platelets.

Washed platelets were adjusted to 5 × 10^7^ /L and incubated at 37 °C for 15 min. For staining, 50 µL of platelet suspension was incubated with 1 µL APC‐conjugated anti‐mouse CD62P antibody (BioLegend, 148303) or APC‐conjugated Annexin V (BioLegend, 640920) at 37 °C in the dark for 30 min. The reaction was terminated by adding 450 µL Tyrode's buffer. Samples were analyzed on a Beckman flow cytometer.

### Serum and Fecal Glutamate Quantification

Mice were sacrificed, and blood was collected by orbital puncture and centrifuged at 3000 rpm for 20 min to obtain serum. For fecal samples, 20 mg of feces were homogenized in 180 µL of physiological saline on ice, followed by centrifugation at 2500 rpm for 10 min. Glutamate concentrations in serum and fecal supernatants were determined using a Glutamate Assay Kit (Nanjing Jiancheng Bioengineering Institute, A074‐1‐2) according to the manufacturer's protocol.

### Pyrolysis‐Gas Chromatography‐Mass Spectrometry (Py‐GC/MS)

Sample pretreatment: (1) Trichloromethane extraction: ≈10 g of trichloromethane solution (for extracting PS, PC, and PMMA) was added to a sample bottle containing 0.5 g of breast tissue. The mixture was ultrasonicated for 10 min, and the supernatant was collected. This process was repeated three times. (2) Hexafluoroisopropyl alcohol extraction: Next, 10 g of hexafluoroisopropyl alcohol (for extracting PET, PA, and PVC) was added to the sample bottle. Ultrasonication was performed for 10 min, and the supernatant was collected, repeating the process three times. (3) Xylene extraction: After hexafluoroisopropyl alcohol extraction, xylene (for extracting PP and PE) was used as the solvent. The sample bottle was placed on a graphite electric heater at 150 °C, and extraction was repeated three times. (4) Heating and concentration: The soluble fractions from the previous extractions were combined and transferred to a new sample bottle. Ultrasonication was performed for 10 min, followed by heating on a graphite electric heating plate at 80 °C for concentration. (5) On‐machine testing: A glass pipette was used to transfer the sample into the injection crucible of the Py‐GC/MS. The sample was dried at 110 °C and analyzed using the Py‐GC/MS system.

Analysis program: Pyrolysis was conducted at 550 °C for 20 min using a PY‐3030D pyrolyzer (Frontier Lab EGA, Japan) coupled with a GCMS‐QP2020 system (Shimadzu, Japan) at a split ratio of 5:1. GC separation was performed with the oven initially held at 40 °C for 2 min, ramped at 20 °C min^−1^ to 320 °C, and held for 14 min. The ion source temperature was set to 230 °C, with a scan range of m/z 29–600. MPs, including PS, PE, PET, SBR, PVC, ABS, N6, PU, PC, and N66, were identified based on characteristic pyrolytic fragments using LaNSolutions software and quantified using calibration curves generated from standard polymers. Method accuracy and precision were verified using certified reference materials.

### Microbiome Analysis

Genomic DNA was extracted from fecal samples using the Fecal Genotype DNA Rapid Extraction Kit (Bioteke, DP4611). The V3‐V4 hypervariable regions of the bacterial 16S rRNA gene were amplified with primers 338F and 806R as previously described. PCR products were separated on a 2% agarose gel, purified using a PCR Clean‐Up Kit (YuHua, Shanghai, China), quantified with a Qubit 4.0 Fluorometer (Thermo Fisher Scientific, USA), pooled in equimolar ratios, and sequenced on an Illumina NextSeq 2000 platform (Illumina, San Diego, USA) by Majorbio Bio‐Pharm Technology Co. Ltd. (Shanghai, China). Primer sequences are provided in Table S1 (Supporting Information).

Data analysis was performed on the Majorbio Cloud Platform (https://cloud.majorbio.com). Alpha diversity indices (Chao1, Shannon) were calculated using mothur (http://www.mothur.org/wiki/Calculators) and compared between groups by the Wilcoxon rank‐sum test. Beta diversity was assessed by principal coordinate analysis (PCoA) based on Bray‐Curtis dissimilarities, with statistical significance determined by PERMANOVA. Differentially abundant taxa (phylum to genus) were identified using LEfSe (LDA score>2, *p*<0.05; http://huttenhower.sph.harvard.edu/LEfSe).

### Untargeted Metabolomics

≈50 mg of fecal material was transferred into a 2 mL centrifuge tube containing a 6 mm stainless steel bead. Metabolites were extracted with 400 µL methanol/water (4:1, v/v) containing 0.02 mg/mL L‐2‐chlorophenylalanine (internal standard). Samples were homogenized in a cryogenic tissue grinder at −10 °C and 50 Hz for 6 min, followed by ultrasonic extraction at 5 °C and 40 kHz for 30 min. After incubation at −20 °C for 30 min, samples were centrifuged at 13000 × g for 15 min at 4 °C. The supernatant was transferred to autosampler vials for LC‐MS analysis.

Metabolomic profiling was performed using an ultra‐high‐performance liquid chromatography system coupled with time‐of‐flight mass spectrometry (UPLC‐TripleTOF, SCIEX; Shanghai Meiji Biomedical Technology Co., Ltd.). Raw data were processed in Progenesis QI (Waters Corp., Milford, USA) for peak detection, alignment, retention time correction, and normalization, generating a data matrix containing retention time, mass‐to‐charge ratio (m/z), and peak intensity. Metabolites were identified by matching against the Human Metabolome Database (HMDB; http://www.hmdb.ca/), Metlin (https://metlin.scripps.edu/), and an in‐house spectral library.

The processed data matrix was analyzed on the Majorbio Cloud Platform (https://cloud.majorbio.com). Principal component analysis (PCA) and orthogonal partial least squares discriminant analysis (OPLS‐DA) were conducted using the “ropls” package (v1.6.2) in R, with model performance assessed by seven‐fold cross‐validation. Metabolites with variable importance in projection (VIP) scores>1.0 and *p*< 0.05 (Student's t‐test) were considered significantly altered. Differential metabolites were mapped to biological pathways using the Kyoto Encyclopedia of Genes and Genomes (KEGG; https://www.kegg.jp/kegg/pathway.html) for functional annotation.

### Data‐Independent Acquisition Mass Spectrometry (DIA‐MS)

Proteomic analysis was conducted by Shanghai Meiji Biomedical Technology Co., Ltd. Following protein extraction, trypsin digestion, and desalting, peptides were reconstituted in MS‐compatible loading buffer and separated using a Vanquish Neo UHPLC system (Thermo Fisher Scientific, USA) equipped with a uPAC High Throughput column (75 µm×5.5 cm, Thermo). The mobile phases consisted of 2% acetonitrile with 0.1% formic acid (A) and 80% acetonitrile with 0.1% formic acid (B), with an 8 min total gradient.

Eluted peptides were analyzed on an Orbitrap Astral mass spectrometer (Thermo Fisher Scientific) operated in DIA mode under positive ionization with an electrospray voltage of 1.5 kV. Full MS scans were acquired over an m/z range of 100–1700. Data acquisition was controlled by Xcalibur 4.7 software (Thermo Fisher Scientific).

Raw DIA data were processed in Spectronaut 19 (Biognosys) for database searching and quantification. Downstream statistical analyses were performed on the Majorbio Cloud Platform. Differentially expressed proteins (DEPs) were identified using unpaired Student's t‐tests with thresholds of *p*< 0.05 and fold change (FC)>1.2. Functional enrichment of DEPs was performed using KEGG pathway analysis, and protein‐protein interaction (PPI) networks were generated via the STRING database (v11.5; https://string‐db.org).

### Cell Viability Assay

4T1 cells were seeded in 96‐well plates at a density of 2000 cells per well and treated with PS‐NPs at final concentrations of 0, 2.5, 5, 10, 20, or 40 µg mL^−1^ for 24, 48, 72, or 96 h. Cell viability was determined using the CCK‐8 (Beyotime, Cat# C0037) according to the manufacturer's instructions.

### Transwell and Wound Healing AssaysTranswell

For transwell assays, the upper chamber of the insert (Corning) was pre‐coated with 50 µL of Matrigel (Corning) and allowed to solidify. 4T1, MDA‐MB‐231, HCC1937 and BT‐549 cells (2 × 10^5^) were seeded in serum‐free medium containing varying concentrations of glutamic acid. Platelets were added to the upper chamber 6 h prior to sample collection. Alternatively, 4T1 cells can be cultured in medium supplemented with different concentrations of PS‐NPs. After 48 h, non‐invaded cells on the upper membrane surface were removed with a cotton swab, while invaded cells on the underside were fixed in methanol and stained with 0.1% crystal violet. Images were acquired, and cell numbers were quantified using Image J software.

For wound healing assays, cells were serum‐starved for 24 h, and a linear scratch was created using a 200 µL pipette tip. Cells were then cultured in medium with or without glutamic acid, and wound closure was monitored every 12 h by microscopy. Alternatively, cells were cultured in medium containing different concentrations of PS‐NPs, and the wound healing was observed under a microscope every 24 h. The percentage of wound closure was quantified using Image J.

### Mammosphere Formation Assay

Single‐cell suspensions were cultured in serum‐free DMEM/F12 supplemented with 1×B27 (Gibco, 12 587 010), 20 ng mL^−1^ epidermal growth factor, 20 ng mL^−1^ basic fibroblast growth factor (Prospec, CYT‐288‐b), 4 µg mL^−1^ insulin, 0.4 ng mL^−1^ hydrocortisone, 0.4% bovine serum albumin (BioFroxx, 4240GR250), and 1% penicillin‐streptomycin. Cultures were maintained in ultra‐low attachment plates at 37 °C in a 5% CO_2_ atmosphere, with medium replenished every 5 days. After 15 days, mammospheres were imaged and quantified by microscopy.

### Statistical Analysis

Statistical analyses were performed using GraphPad Prism version 10 (GraphPad Software, USA). Data are presented as mean±standard error of the mean (SEM). Comparisons between two groups were assessed using unpaired two‐tailed Student's t‐tests, and multiple group comparisons were analyzed by one‐way analysis of variance (ANOVA). Statistical significance was defined as *p* < 0.05 (*), *p* < 0.01 (**), *p* < 0.001 (***), and *p* < 0.0001 (****).

## Conflict of Interest

The authors declare no conflict of interest.

## Author Contributions

Y.S. and Z.D. conceptualized and directed the study. L.Z., P.X. and Y.S. performed all experiments and analyzed data. M.Z., K.L., S.T., X.F., J.L. and B.Y. helped with the experiments. L.Z., P.X. and Y.S. wrote the manuscript with input from all authors.

## Supporting information



Supporting Information

## Data Availability

The data that support the findings of this study are available in the supplementary material of this article.
