# Supplementary material for: Synergistic Promotion of Triple‐Negative Breast Cancer Tumorigenesis and Metastasis by Oral Polystyrene Nanoplastics Exposure via Alloprevotella‐Derived Glutamate and Platelet Activation
Source: Adv Sci (Weinh). 2025 Sep 24;12(46):e08310. doi: 10.1002/advs.202508310 (PMC12697842; doi:10.1002/advs.202508310)
Supplement: Supplementary file 1 — Supporting Information [file ADVS-12-e08310-s001.docx]

Supporting Information

**Synergistic promotion of triple-negative breast cancer tumorigenesis and metastasis by oral polystyrene nanoplastics exposure via *Alloprevotella*-derived glutamate and platelet activation**

Leilei Zhu^1#^, Peihao Xu^1#^, Mingyuan Zhou^1^, Keiwei Li^1^, Shasha Tian^2^, Xuemei Fan^3^, Junling Liu^3^, Baodong Ye^4^, Zhishan Ding^1*^, Yingzhi Shen^1,5*^*


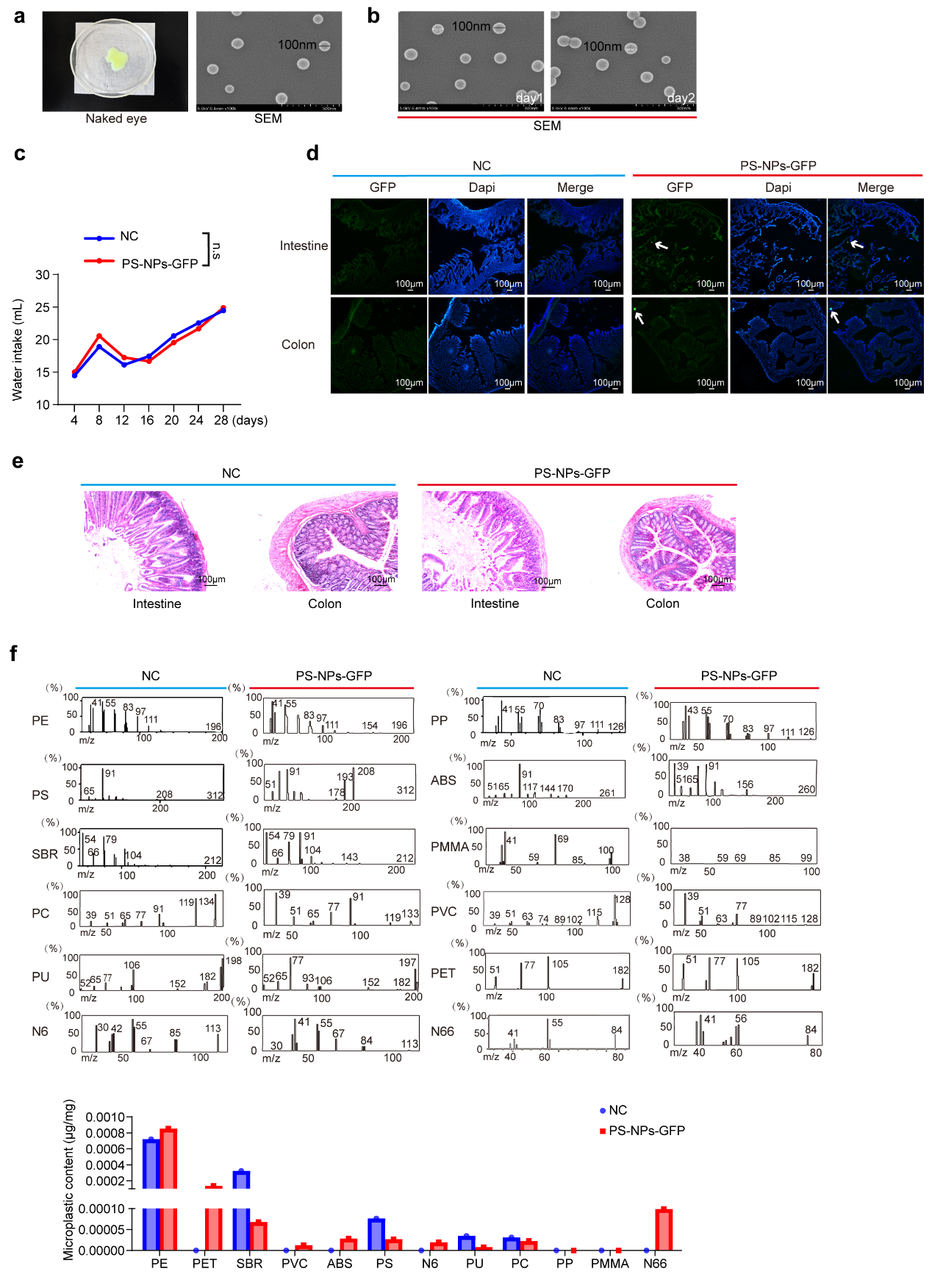


**Figure S1.** (Oral exposure to PS-NPs resulted in intestinal accumulation without significant tissue damage or systemic acccumulation.)

**a,** Gross morphology and scanning electron microscope (SEM) images of polystyrene nanoparticles (PS-NPs).

**b,** SEM images demonstrating the distribution and stability of PS-NPs in aqueous solution over 2 days.

**c,** Daily water intake of mice during 28-day exposure. No significant difference was observed between PS-NPs-exposed and control groups (n = 5 per group).

**d,** Confocal microscopy showing localization of fluorescently labeled PS-NPs (green) in intestine and colon tissue of tumorigenesis group mice. Nuclei stained with DAPI (blue). White arrows indicated PS-NPs (n = 5 per group).

**e,** Representative hematoxylin and eosin (H&E)-stained sections of intestinal and colon tissues from tumorigenesis group at endpoint (n = 5 per group).

**f,** Pyrolysis-gas chromatography-mass spectrometry (Py-GC-MS) was used to detect 12 common microplastics in pooled breast tumor tissue samples from PS-NPs and control groups (n = 5 per group). The detection limit was 0.001 ppm. Representative peak and quantification profiles are shown for each group.

Statistical analysis was performed using unpaired two-tailed t-tests for panel **c**. n.s, not significant.


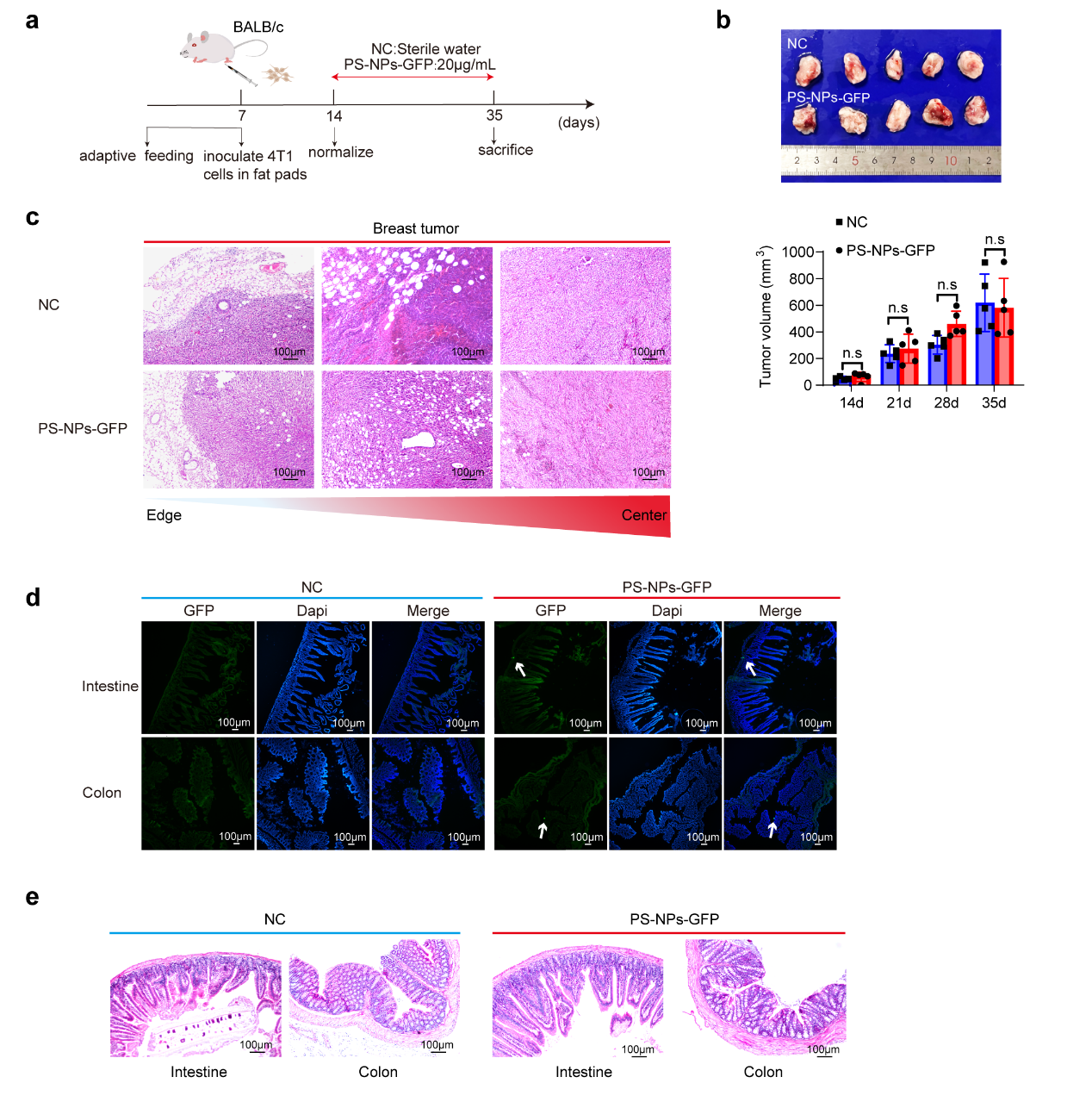


**Figure S2.** (Oral exposure to PS-NPs induced in intestinal accumulation without significant tissue damage or tumor growth effects.)

**a,** Experimental timeline of tumor growth model. Seven-week-old BALB/c mice received PS-NPs-GFP (20 μg/mL) in drinking water for 21 days. On day 7, 4T1 cells were orthotopically injected into the mammary fat pad. Tumor volumes were normalized on day 14, and mice were sacrificed on day 35 (n = 5 per group).

**b,** Representative gross images of mammary tumors from control (NC) and PS-NPs-GFP groups. Tumor volumes per mouse were shown as mean ± SEM (n = 5 per group).

**c,** Representative H&E-stained mammary tumor sections at endpoint (n = 5 per group).

**d,** Confocal images of PS-NPs-GFP (green) in intestine and colon tissue; nuclei stained with DAPI (blue). White arrows indicate PS-NPs (n = 5 per group).

**e,** Representative H&E-stained sections of intestine and colon at endpoint.

Statistical analysis was performed using unpaired two-tailed t-tests for panel **b**. n.s, not significant.


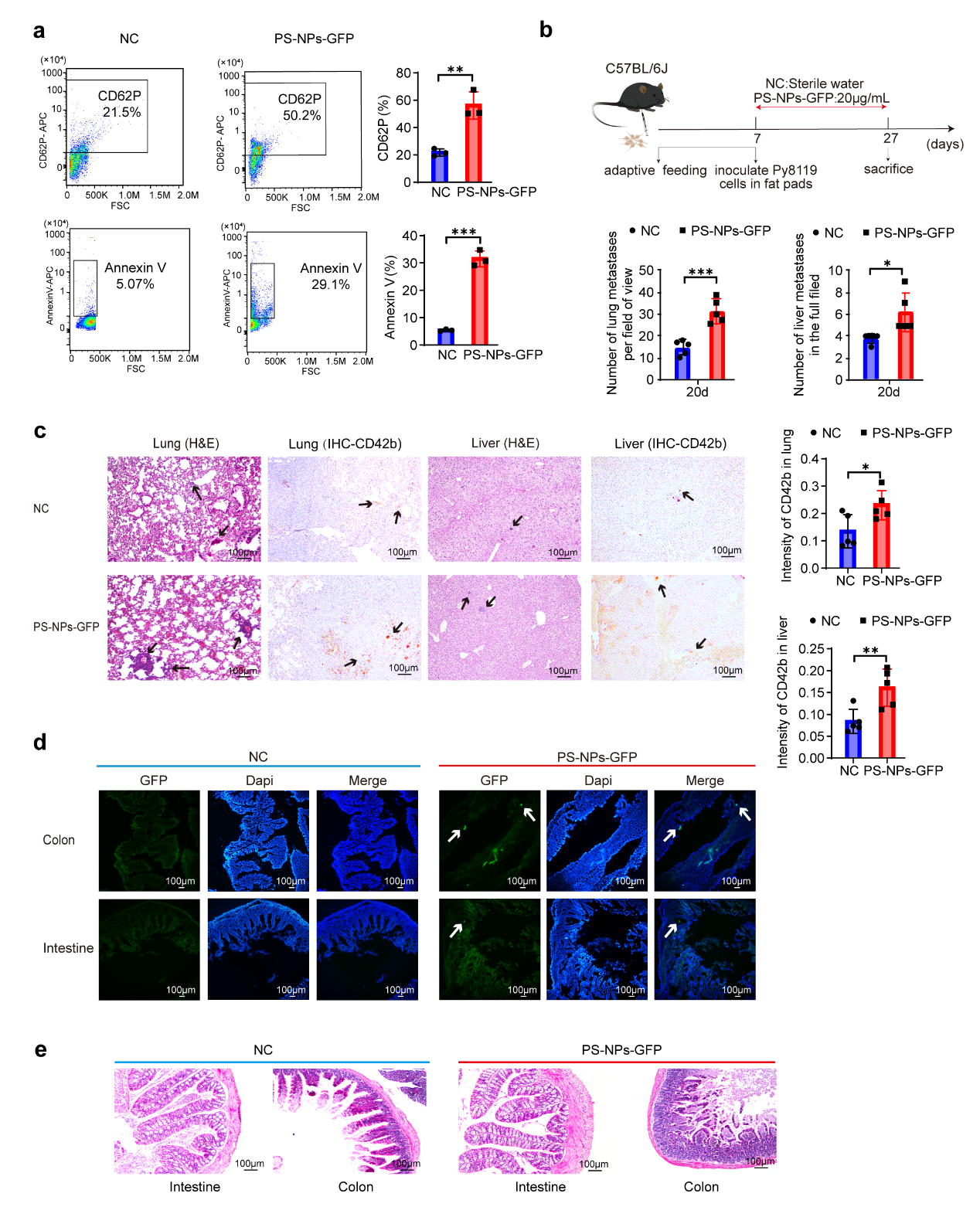


**Figure S3.** (Oral exposure to PS-NPs promoted breast cancer metastasis in Py8119 tumor-bearing mice and resulted in intestinal accumulation without overt tissue damage.)

**a,** Flow cytometry analysis of platelet activation. Representative plots showed Annexin V and CD62P staining on platelets isolated from NC and PS-NPs-GFP groups in the metastasis model (n = 3 per group). Platelets were gated by forward and side scatter and stained with fluorochrome-conjugated anti-Annexin V and anti-CD62P antibodies. Percentages of positive cells were indicated. Data represented at least three independent experiments.

**b,** Schematic of experimental design. Seven-week-old C57BL/6J mice were administered PS-NPs-GFP (20 μg/mL) in drinking water ad libitum, followed by orthotopic injection of Py8119 cells into the mammary fat pad. Mice were sacrificed 20 days post-injection (n = 5 per group).

**c,** Representative H&E-stained sections of lung, and liver at endpoint. Metastatic foci were quantified in lungs and livers based on H&E staining; immunohistochemical analysis of CD42b was performed on tissue microarrays from lung and liver samples, with quantification presented (mean ± SEM; n = 5 per group).

**d**, Confocal microscopy images showing distribution of fluorescently PS-NPs (green) in colon and intestine tissue of metastasis group mice. Nuclei were stained (blue). White arrows indicated PS-NPs (n = 5 per group).

**e,** Representative H&E-stained sections of intestine and colon from metastasis group mice at endpoint (n = 5 per group). No significant tissue damage was observed.

Statistical analysis was performed using unpaired two-tailed t-tests for panel **a-c**. * *p* < 0.05; ** *p* < 0.01; *** *p* < 0.001.


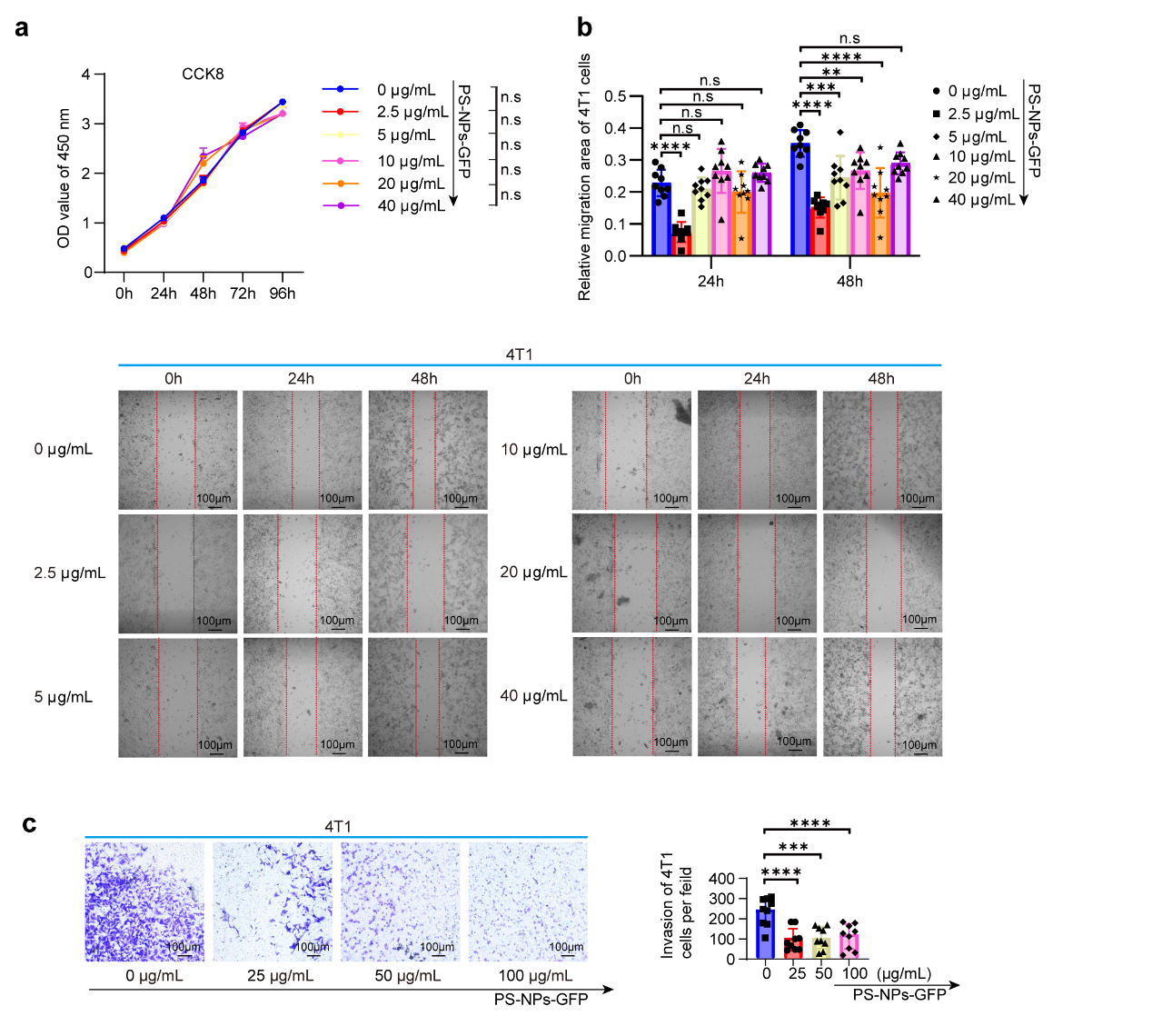


**Figure S4.** (Direct *in vitro* exposure to PS-NPs did not significantly affect 4T1 cell proliferation, migration, or invasion.)

**a,** 4T1 cells were treated with increasing concentrations of PS-NPs (0, 2.5, 5, 10, 20, and 40 μg/mL) for 96 hours. Cell viability was assessed using the CCK-8 assay, with absorbance at 450 nm normalized to untreated controls. Data presented as mean ± SEM from three independent experiments (n =3).

**b,** Wound healing assay assessing migration of 4T1 cells treated with PS-NPs (0, 2.5, 5, 10, 20, and 40 μg/mL). Representative images were captured at 0, 24, and 48 h post-scratch. Wound closure was quantified using Image J and expressed as a percentage relative to 0 h. Data represented as mean ± SEM from three independent experiments (n = 3).

**c,** Transwell migration assay of 4T1 cells treated with PS-NPs (0, 25, 50, and 100 μg/mL) for 24 hours. Invaded cells were stained with crystal violet and quantified from three randomly selected fields per well. Representative images and quantification were shown (n = 3 independent experiments).

Statistical analysis was performed using one-way ANOVA with Dunnett’s post hoc test for panels **a-c**. ** *p* < 0.01; *** *p* < 0.001; **** *p* < 0.0001; n.s, not significant.


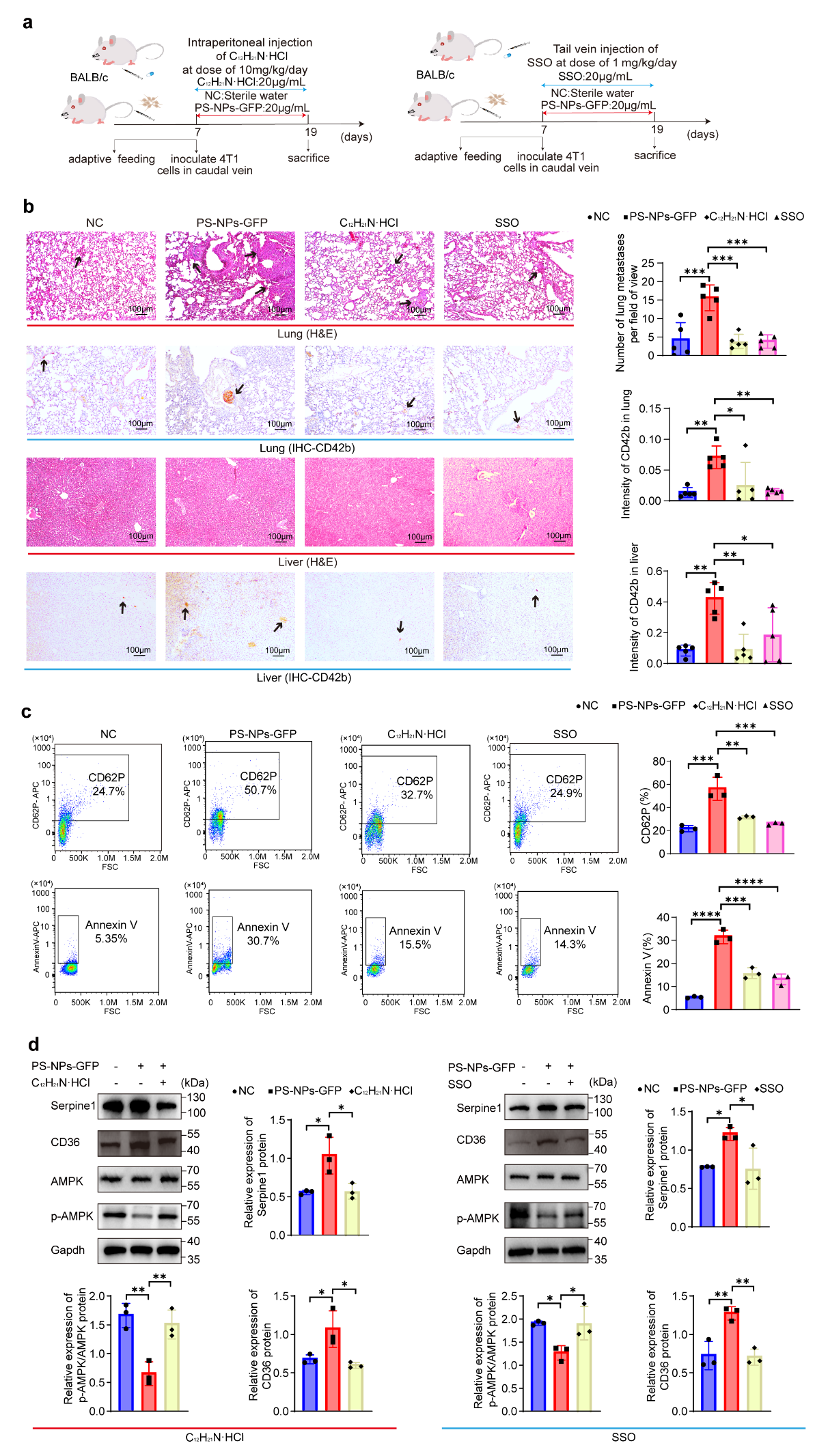


**Figure S5.** (C₁₂H₂₁N·HCl and SSO mitigated PS-NPs-induced metastasis, platelet activation, and AMPK-related signaling in 4T1 tumor-bearing mice.)

**a,** Experimental design schematic. Seven-week-old BALB/c mice received PS-NPs-GFP (20 μg/mL) via drinking water ad libitum, followed by caudal vein injection of 4T1 cells. The C₁₂H₂₁N·HCl group received daily intraperitoneal injections of 10 mg/kg C₁₂H₂₁N·HCl, while the SSO group was administered 1 mg/kg SSO via daily tail vein injection. Mice were sacrificed 12 days post-injection (n = 5 per group).

**b,** Quantification of metastatic foci in lungs based on H&E staining. Representative H&E-stained liver sections were shown. Representative immunohistochemical images of lung and liver stained for CD42b with corresponding quantification of staining scores (mean ± SEM, n = 5 mice per group).

**c,** Flow cytometry analysis of platelet activation. Representative plots of Annexin V and CD62P staining on platelets isolated from NC, PS-NPs-GFP, C₁₂H₂₁N·HCl, and SSO groups (n = 3 per group). Platelets were gated based on forward and side scatter, and stained with fluorochrome-conjugated anti-Annexin V and anti-CD62P antibodies. Percentages of positive cells were indicated. Data represented at least three independent experiments.

**d,** Western blot analysis of platelet AMPK pathway proteins, Serpine, and CD36 from NC, PS-NPs-GFP, C₁₂H₂₁N·HCl, and SSO groups. Pepresentative blots and densitometric quantification normalized to Gapdh were shown (mean ± SEM; n = 3 per group).

Statistical analysis was performed using one-way ANOVA with Dunnett’s post hoc test for panels **b-d**. * *p* < 0.05; ** *p* < 0.01; *** *p* < 0.001; **** *p* < 0.0001.


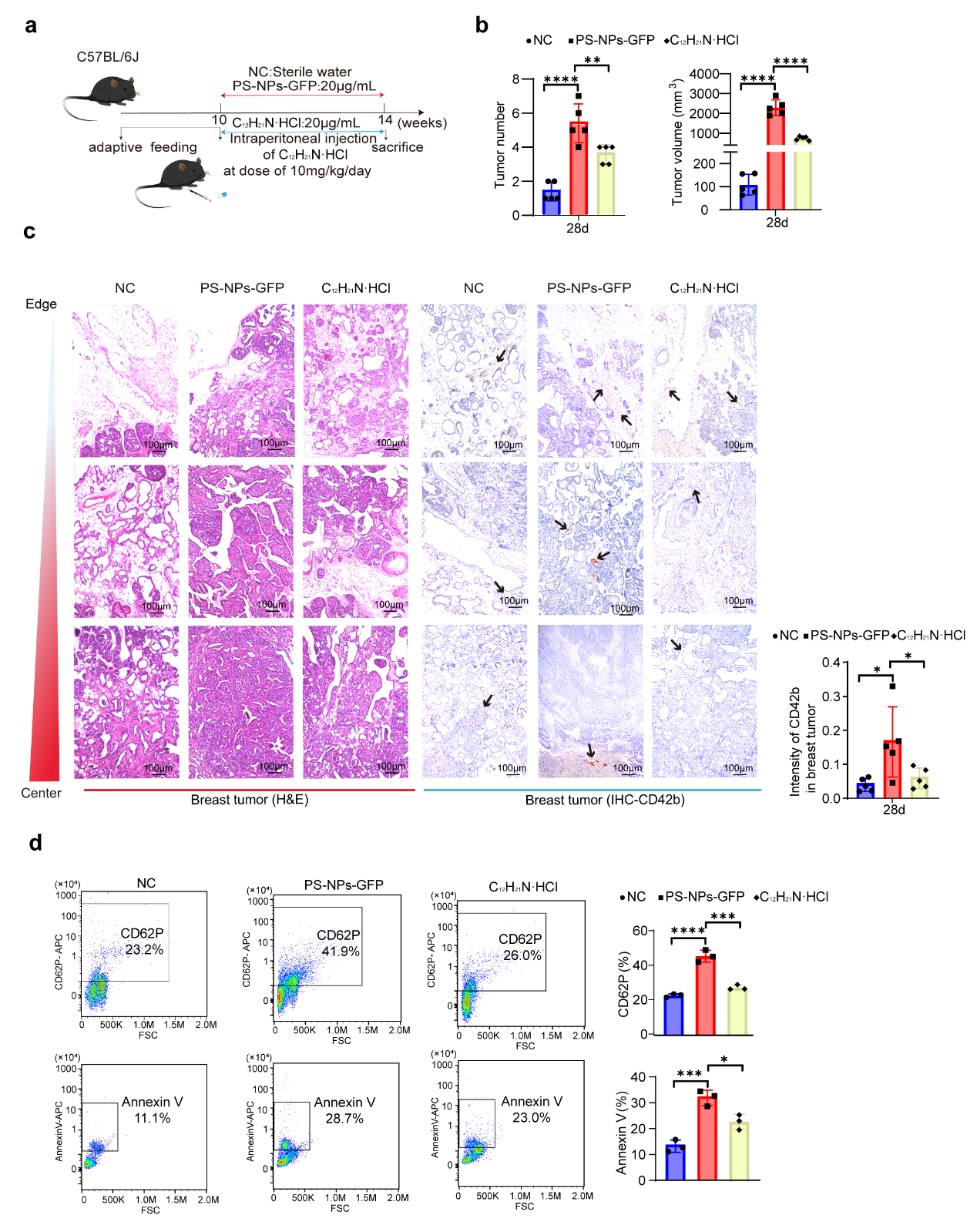


**Figure S6.** (C₁₂H₂₁N·HCl attenuated PS-NPs-induced tumorigenesis and platelet activation in *MMTV-PyMT* transgenic mice.)

**a,** Experimental design schematic. Ten-week-old *MMTV-PyMT* transgenic mice received PS-NPs-GFP (20 μg/mL) in drinking water ad libitum. The C₁₂H₂₁N·HCl group was administered daily intraperitoneal injections of 10 mg/kg C₁₂H₂₁N·HCl. Mice were sacrificed 4 weeks post-injection (n = 5 per group).

**b,** Quantification of tumor number and volume per mouse revealed a significant increase in the PS-NPs-GFP group compared to NC and C₁₂H₂₁N·HCl groups (mean ± SEM; n = 5 per group).

**c,** Representative H&E-stained mammary tissue sections at endpoint. Immunohistochemical analysis of breast tumor tissue microarrays stained for CD42b, with quantification of staining scores (mean ± SEM; n = 5 mice per group).

**d,** Flow cytometry analysis of platelet activation. Representative plots of Annexin V and CD62P staining on platelets isolated from NC, PS-NPs-GFP, and C₁₂H₂₁N·HCl groups (n = 3). Platelets were gated by forward and side scatter and stained with fluorochrome-conjugated antibodies. Percentages of positive cells were indicated. Data represented at least three independent experiments.

Statistical analysis was performed using one-way ANOVA with Dunnett’s post hoc test for panels **b-d**. * *p* < 0.05; ** *p* < 0.01; *** *p* < 0.001; **** *p* < 0.0001.


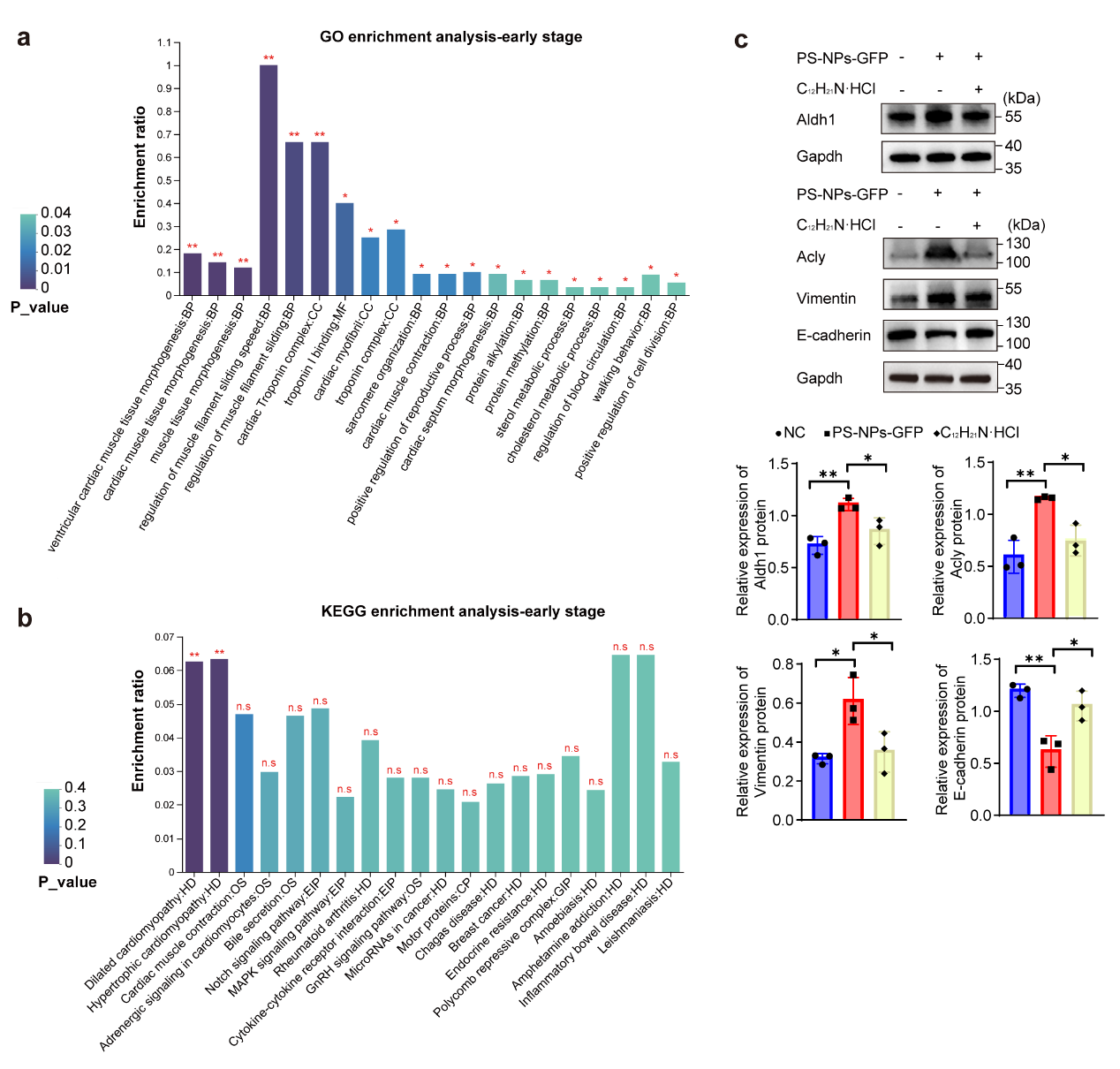


**Figure S7.** (GO and KEGG enrichment analyses of differentially expressed proteins in early-stage mammary tumors following oral PS-NPs exposure, and expression of Aldh1, Acly, EMT-related proteins in breast tumor tissue in response to PS-NPs-GFP and C₁₂H₂₁N·HCl treatment.)

**a,** Gene Ontology (GO) enrichment analysis of the top 30 differentially expressed proteins, highlighting representative terms in biological process (BP), molecular function (MF), and cellular component (CC) categories (n = 5 per group).

**b,** Kyoto Encyclopedia of Genes and Genomes (KEGG) pathway enrichment analysis of the top 30 differentially expressed proteins. The top enriched pathways were shown (n = 5 per group).

**c,** Western blot validation of Aldh1, Acly, E-cadherin, and Vimentin expression (n = 3 per group) with Gapdh as loading control. Densitometric quantification was performed using Image J, and data were normalized to internal controls (mean ± SEM).

Statistical analysis was performed using unpaired two-tailed t-tests for panel **a, b**; differentially expressed proteins (DEPs) were defined by fold change > 1.2 or < 0.83 and P_value < 0.05. One-way ANOVA with Dunnett’s post hoc test for panels **c**. * *p* < 0.05; ** *p* < 0.01; n.s, not significant.


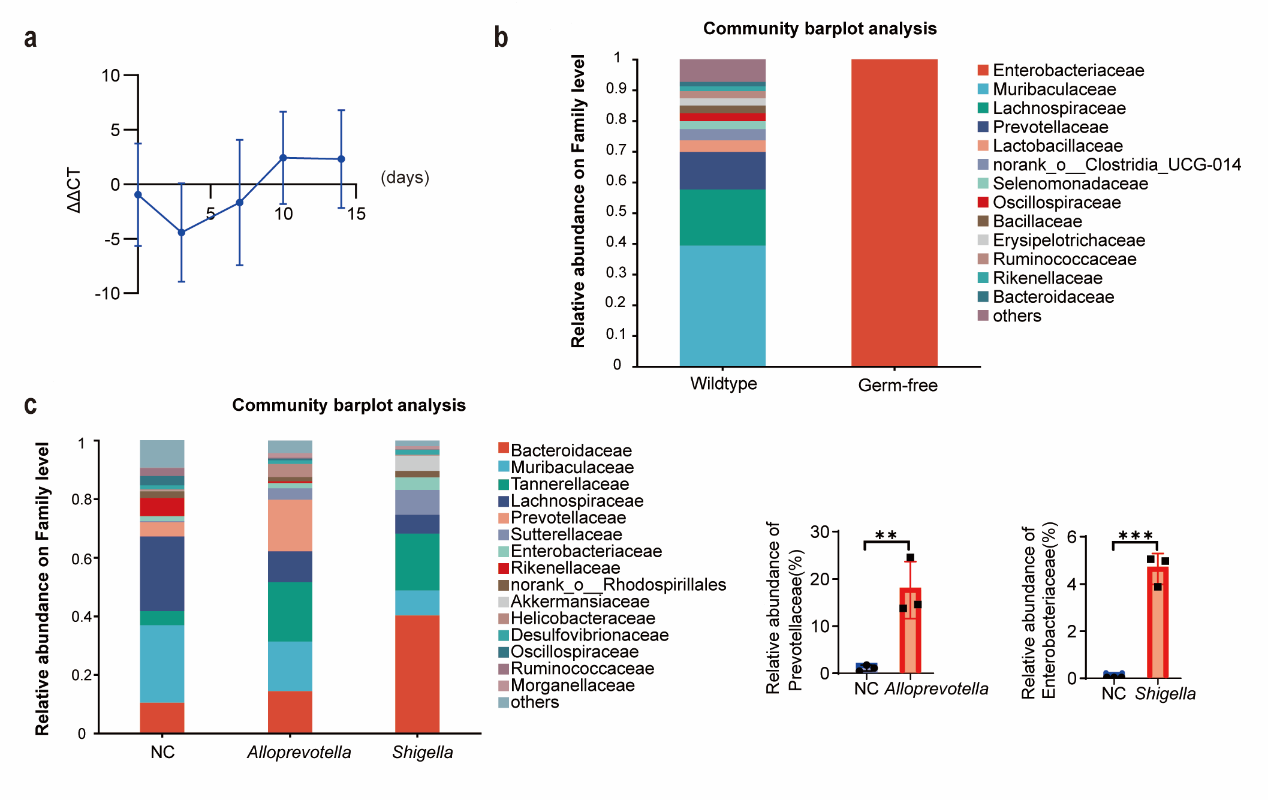


**Figure S8.** (Establishment of pseudo-germ-free and targeted microbiota-transplanted mouse models.)

**a,** Optimization of the pseudo-germ-free model. Mice were treated with a broad-spectrum antibiotic cocktail via oral gavage for 10 consecutive days. Fecal microbiota diversity was quantified by qPCR, showing stable minimal microbiota levels after 10 days (n = 3).

**b,** Relative abundance of gut microbiota at the family level in pseudo-germ-free and conventional mice (n = 3 per group).

**c,** Relative abundance of gut microbiota at the family level in mice colonized with *Alloprevotella*-enriched, *Shigella*-enriched, and conventional microbiota (n = 3 per group). Changes in Prevotellaceae (family of *Alloprevotell*a) and Enterobacteriaceae (family of *Shigella*) were shown.

Statistical analysis in **c** was performed using unpaired two-tailed t-tests. ** *p* < 0.01; *** *p* < 0.001.


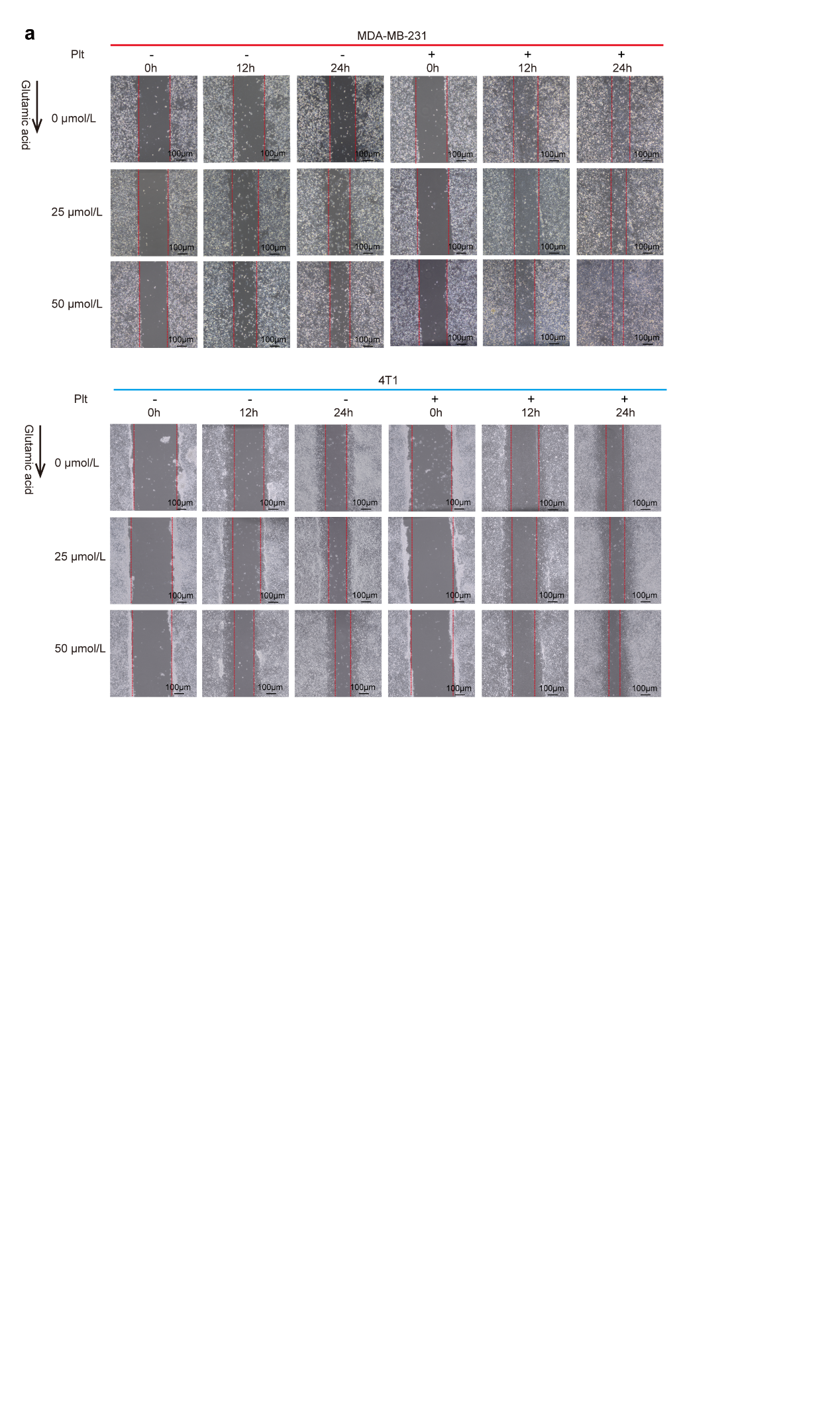


**Figure S9.** (Raw images of wound healing assays in MDA-MB-231 and 4T1 cells.)

**a,** Representative raw images from wound healing assays in MDA-MB-231 and 4T1 cells.


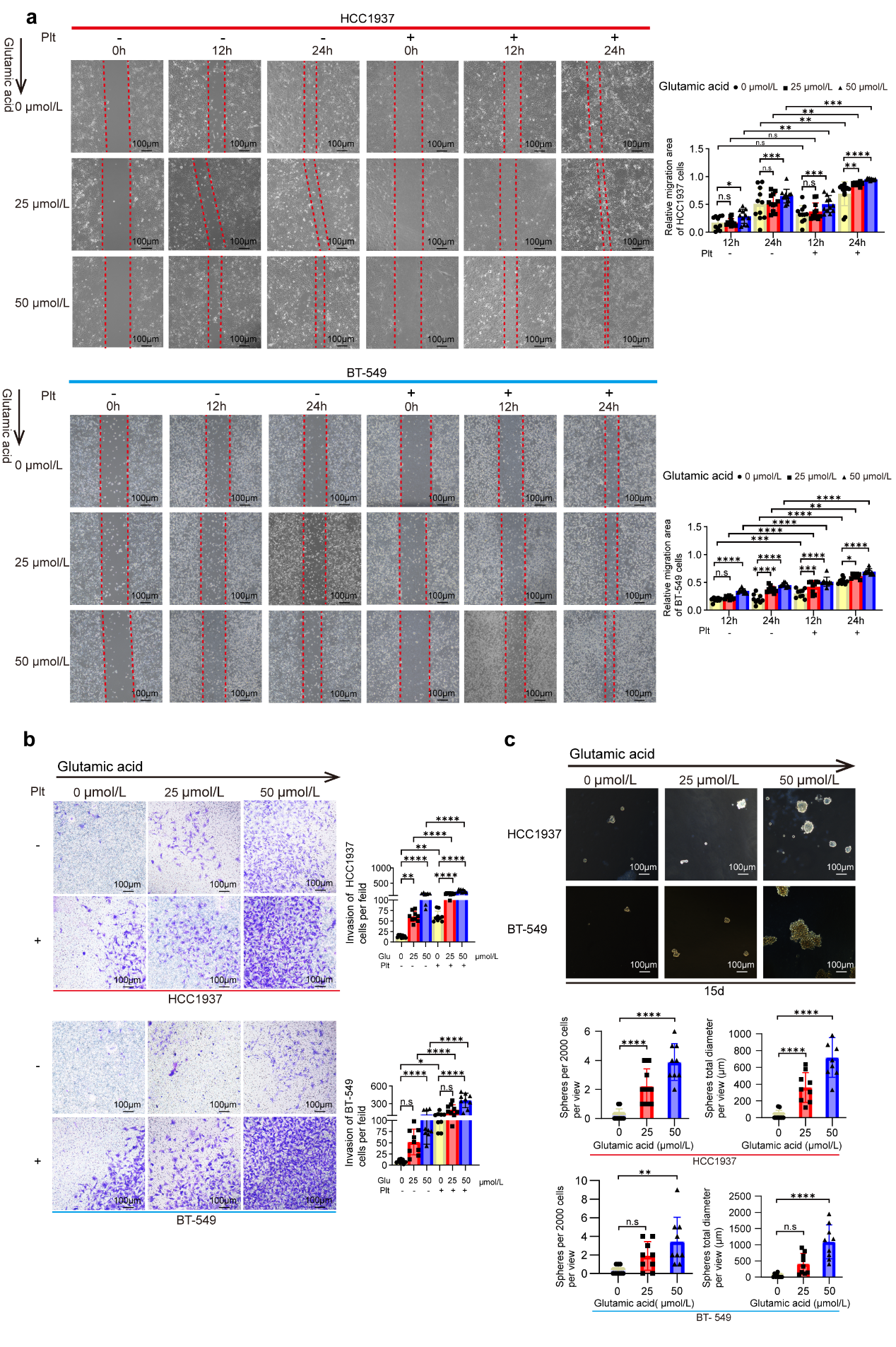


**Figure S10.** (Glutamate and platelet stimulation promoted migratory, invasive, and stem-like phenotypes in TNBC cells.)

**a,** Wound healing assay of HCC1937 and BT-549 cells treated with increasing concentrations of glutamate (0, 25, 50 μmol/L) and/or platelets. Representative images were captured at 0, 12 and 24 h post-scratch. Wound closure was quantified using Image J and presented as mean ± SEM (n = 3 independent experiments).

**b,** Transwell assays of HCC1937 and BT-549 cells treated with glutamate (0, 25, 50 μmol/L) and/or platelets. Invaded cells were stained with crystal violet and quantified in three randomly selected fields per well. Data were presented as mean ± SEM (n = 3 independent experiments).

**c,** Quantification of tumor spheroids (>50 μm) formed by HCC1937 and BT-549 cells following glutamate (0, 25, 50 μmol/L) treatment. Number and diameter of spheroids were shown (n = 3 independent experiments).

Statistical analysis was performed using one-way ANOVA with Dunnett’s post hoc test and unpaired two-tailed t-tests for panels **a, b**; and one-way ANOVA with Dunnett’s post hoc test for panels **c**. * *p* < 0.05; ** *p* < 0.01; *** *p* < 0.001; **** *p* < 0.0001; n.s, not significant.

**
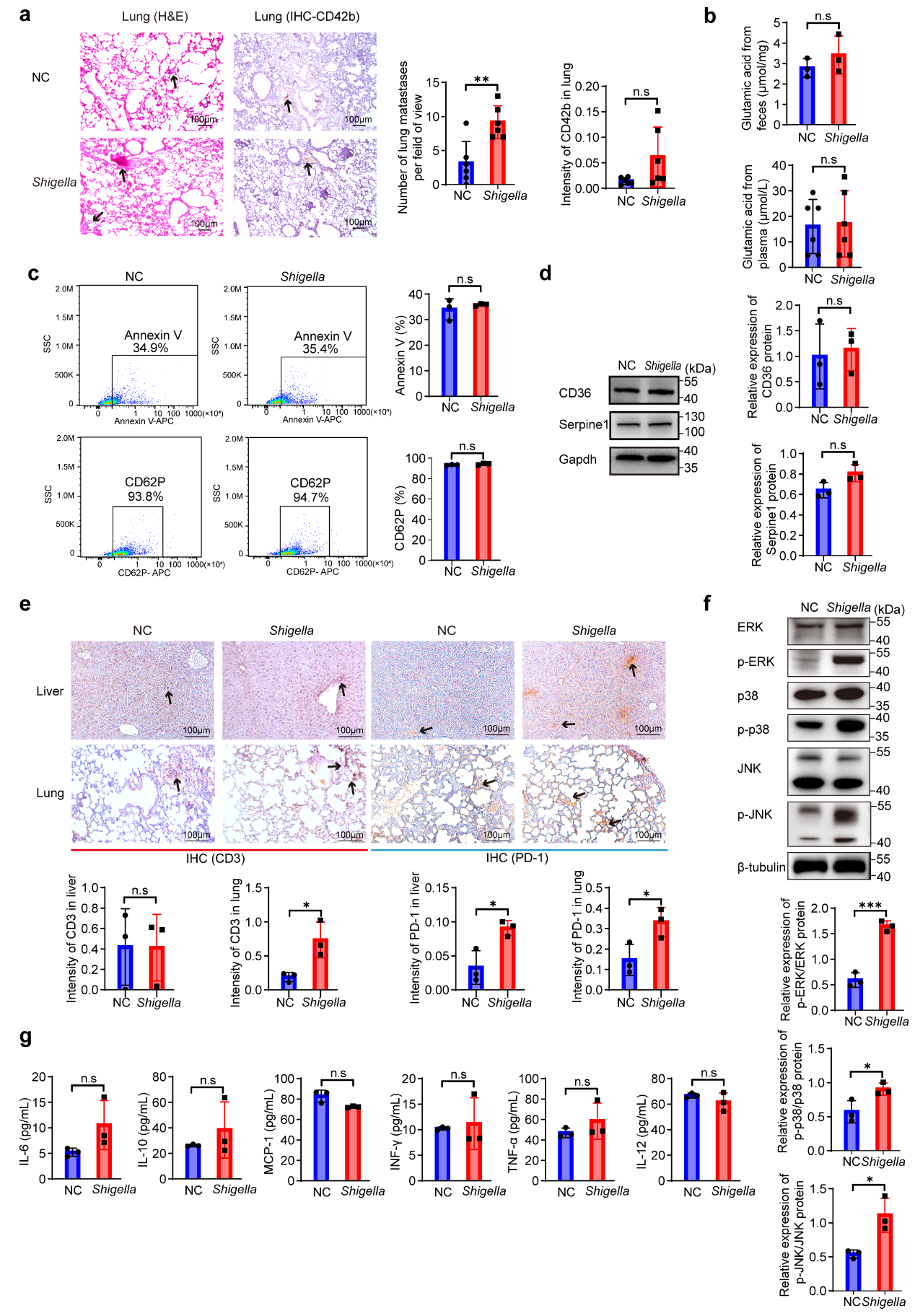
**

**Figure S11.** (*Shigella* facilitated TNBC metastasis independently of glutamate and platelet activation, and was associated with localized inflammation and MAPK signaling pathway.)

**a,** Quantification of pulmonary metastatic foci in 4T1 tumor-bearing mice based on H&E staining. Representative immunohistochemical images of lung tissue microarrays stained for CD42b, with quantification of staining scores (mean ± SEM; n = 6 mice per group).

**b,** Glutamate concentrations in feces (n = 3 per group) and serum (n = 6 per group) of NC and *Shigella* groups during metastasis (mean ± SEM).

**c,** Flow cytometry analysis of platelet activation. Representative plots showing Annexin V and CD62P staining on platelets isolated from peripheral blood of NC and *Shigella* groups during tumorigenesis and metastasis (mean ± SEM; n = 3 per group). Platelets were gated by forward and side scatter and stained with fluorochrome-conjugated antibodies. Percentages of positive cells were indicated. Data represented at least three independent experiments.

**d,** Western blot analysis of CD36 and Serpine1 expression in NC and *Shigella* groups, with Gapdh as loading control. Representative blots and densitometric quantification relative to Gapdh were shown (mean ± SEM; n = 3 per group).

**e,** Representative IHC images of lung and liver tissues stained for CD3 and PD-1, with quantification of staining scores (mean ± SEM; n = 3 mice per group).

**f,** Western blot analysis of MAPK pathway proteins in tumor tissues from NC and *Shigella* groups. Representative blots and densitometric quantification normalized to β-tubulin as loading control were shown (mean ± SEM; n = 3 per group).

**g.** Flow cytometry analysis of serum inflammatory cytokines IL-6, MCP-1, IL-12, TNF-α, IFN-γ, and IL-10 in NC and *Shigella* groups (mean ± SEM; n = 3 per group).

Statistical analysis was performed using unpaired two-tailed t-tests for panels **a-g**. * *p* < 0.05; ** *p* < 0.01; *** *p* < 0.001; n.s, not significant.

| **Name** | **Sequences 5’-3’** |
| --- | --- |
| Fas-F | GGTTACACCTGTGCTAGGTGTTG |
| Fas-R | TCAGGCGCATGAGGCTCAGC |
| PPARγ-F | CTGACCCAATGGTTCTGAT |
| PPARγ-R | GGTGGAGATGCAGGTTCTAC |
| Chrebp3-F | GGGACAAGATCCGGCTGAA |
| Chrebp3-R | GCTCTTCCTCCGTTGCACAT |
| Gapdh-F | AGGTCGGTGTGAACGGATTTG |
| Gapdh-R | GGGGTCGTTGATGGCAACA |
| 16-S diversity-F | 338F-ACTCCTACGGAGCAGCAG |
| 16-S diversity-R | 806R-GGACTACHVGGGTWTCAAT |
| 16S-F | CCTACGGGNGCWGCAG |
| 16S-R | GACTACHVGGGTATCTAATCC |
| PyMT-1 | GGAAGCAAGTACTTCACAAGGG |
| PyMT-2 | GGAAAGTCACTAGGAGCAGGG |
| PyMT-3 | CAAATGTTGCTTGTCTGGTG |
| PyMT-4 | GTCAGTCGAGTGCACAGTTT |

**Supplementary Table 1.** (Sequences of PCR primers used in this study.)

F, forward; R, reverse.
